# Tamsulosin Deprescribing for Lower Urinary Tract Symptoms in Older Men: A Randomized Clinical Trial

**DOI:** 10.1001/jamanetworkopen.2026.21639

**Published:** 2026-07-06

**Authors:** Scott R. Bauer, Stacey A. Kenfield, Akinyemi Oni-Orisan, Michael G. Shlipak, Kaiwei Lu, Natalie Rios, Robert Pearce, Joseph Harmon, Charles E. McCulloch, Liusheng Huang, Michael A. Steinman, Benjamin N. Breyer

**Affiliations:** 1 Division of General Internal Medicine, Department of Medicine, University of California, San Francisco; 2 Department of Urology, University of California, San Francisco; 3 San Francisco VA Healthcare System, San Francisco, California; 4 Department of Epidemiology and Biostatistics, University of California, San Francisco; 5 Department of Clinical Pharmacy, University of California, San Francisco; 6 Division of Geriatrics, Department of Medicine, University of California, San Francisco

## Abstract

**Importance:**

Tamsulosin is the most common treatment for lower urinary tract symptoms (LUTS) attributed to benign prostatic hyperplasia (BPH). The ability of N-of-1 deprescribing trials to identify who may benefit from discontinuing chronic tamsulosin therapy is unknown.

**Objective:**

To determine if N-of-1 deprescribing trials can identify older men who are receiving minimal benefit from tamsulosin therapy for LUTS.

**Design, Setting, and Participants:**

Double-blind, placebo-controlled, multiple crossover (N-of-1) randomized clinical trial conducted at a single academic urology department between September 2021 and July 2022. After a 1-week placebo run-in, men aged 55 to 80 years who were continuous users of tamsulosin for at least 12 months to treat symptoms of BPH were randomly assigned to 2 blocks of 2-week treatment periods alternating tamsulosin and placebo, separated by 1-week washout periods. Data were analyzed from November 2022 to October 2024.

**Intervention:**

Tamsulosin, dosed at either 0.4 mg or 0.8 mg, vs matching placebo.

**Main Outcomess and Measures:**

The efficacy outcome was daily American Urological Association Symptom Index (AUASI) with an adapted recall period of 24 hours. Effect size was categorized based on the upper bound of the individual 95% CI for the tamsulosin-placebo difference in daily AUASI: minimal or no (≥0), moderate (>−6.0 to <0), or strong (≤−6.0) effect. Adverse drug events were assessed daily as a secondary outcome. Demographics, clinical characteristics, and attitudes toward deprescribing were assessed at baseline.

**Results:**

Among the 31 participants enrolled, the mean (SD) age was 68.8 (5.7) years. Of 30 participants who attempted the full N-of-1 protocol, 11 (36.7%) had minimal or no tamsulosin effect, 11 (36.7%) had moderate effect, 4 (13.3%) had strong effect, and 4 (13.3%) did not tolerate the 1-week placebo run-in due to worsening symptoms. Based on a mean (SD) of 54 (3) daily LUTS assessments per participant, individual-level estimated mean difference in daily AUASI between tamsulosin and placebo ranged from −10.9 (95% CI, −12.6 to −9.1) to 2.1 (95% CI, 0.4 to 3.9). The group-level mean difference in daily AUASI was −2.96 (95% CI, −4.37 to −1.54).

**Conclusions and Relevance:**

In this randomized N-of-1 clinical trial of tamsulosin deprescribing, treatment response was heterogeneous. The findings suggest that 1 in 3 older men who had minimal symptomatic benefit from tamsulosin may be high-priority candidates for deprescribing.

**Trial Registration:**

ClinicalTrials.gov Identifier: NCT05415748

## Key Points

**Question:**

Can placebo-controlled N-of-1 deprescribing trials accurately quantify the individualized benefits and harms of tamsulosin therapy for older men with lower urinary tract symptoms?

**Findings:**

In this crossover trial, among 30 participants who attempted the N-of-1 protocol, 11 (36.7%) demonstrated minimal or no effect of tamsulosin, 11 (36.7%) demonstrated moderate effect, 4 (13.3%) demonstrated a strong effect, and 4 (13.3%) did not tolerate the placebo run-in.

**Meaning:**

In this proof-of concept randomized N-of-1 clinical trial of tamsulosin deprescribing, treatment response was highly heterogeneous and approximately 1 in 3 participants receiving tamsulosin therapy for benign prostatic hyperplasia had minimal or no effect of tamsulosin vs placebo on urinary symptoms.

## Teaser

This crossover trial evaluates whether N-of-1 deprescribing trials can identify older men who are receiving minimal benefit from tamsulosin therapy for lower urinary tract symptoms.

## Introduction

One in 3 men will be affected by moderate-to-severe lower urinary tract symptoms (LUTS) in their lifetime.^1^ These common symptoms are significant contributors to worse health-related quality of life^2,3,4,5,6,7^ and are risk factors for several important geriatric outcomes: new mobility impairment, falls, fractures, disability, and death.^8,9,10,11,12^ In the absence of widely available and easily interpretable diagnostic tests, the majority of male LUTS are attributed to benign prostatic hyperplasia (BPH) and are treated empirically with prostate-specific therapies, which are often continued until an adverse event or death.^1^

α-1 Adrenergic receptor antagonists (α1-blockers), particularly tamsulosin, are the most commonly prescribed urologic medications; 1 in 5 men with LUTS are prescribed tamsulosin.^13,14^ The prevalence of α1-blocker therapy is particularly high among older men because they are the most likely to develop LUTS and the least likely to discontinue medications once initiated, even after BPH surgery.^13,15,16^ Despite widespread use, the efficacy of α1-blockers is modest, and many men who discontinue α1-blocker therapy do not require additional treatment.^17,18,19,20^ In addition to modest benefits, the harms of α1-blockers, such as orthostatic hypotension, dizziness, falls, and fractures, have led to recommendations that they be used with caution in older men.^14,21^ Furthermore, the natural history of LUTS is dynamic and includes spontaneous resolution in a subset of men.^22^ Given the lack of availability of urodynamics and other potential predictors of tamsulosin treatment response in primary care settings, N-of-1 trials during α1-blocker therapy initiation or discontinuation may be a practical approach to achieve personalized and patient-centric LUTS care for older men.

To address this gap in knowledge, we performed a series of double-blind, placebo-controlled N-of-1 deprescribing trials, or multiple crossover trials, to determine the efficacy of tamsulosin compared with placebo among older men who were receiving chronic tamsulosin therapy from a urologist to treat symptoms of BPH. The objective of this proof-of-concept study was to determine if placebo-controlled N-of-1 deprescribing trials can identify older men who are likely to benefit from stopping ineffective chronic tamsulosin therapy for LUTS. We hypothesized that daily symptom assessments for 12 weeks during randomly allocated placebo and tamsulosin treatment periods is sufficient to quantify the individualized benefits and harms of chronic tamsulosin therapy.

## Methods

### Study Design and Setting

**Figure 1.**
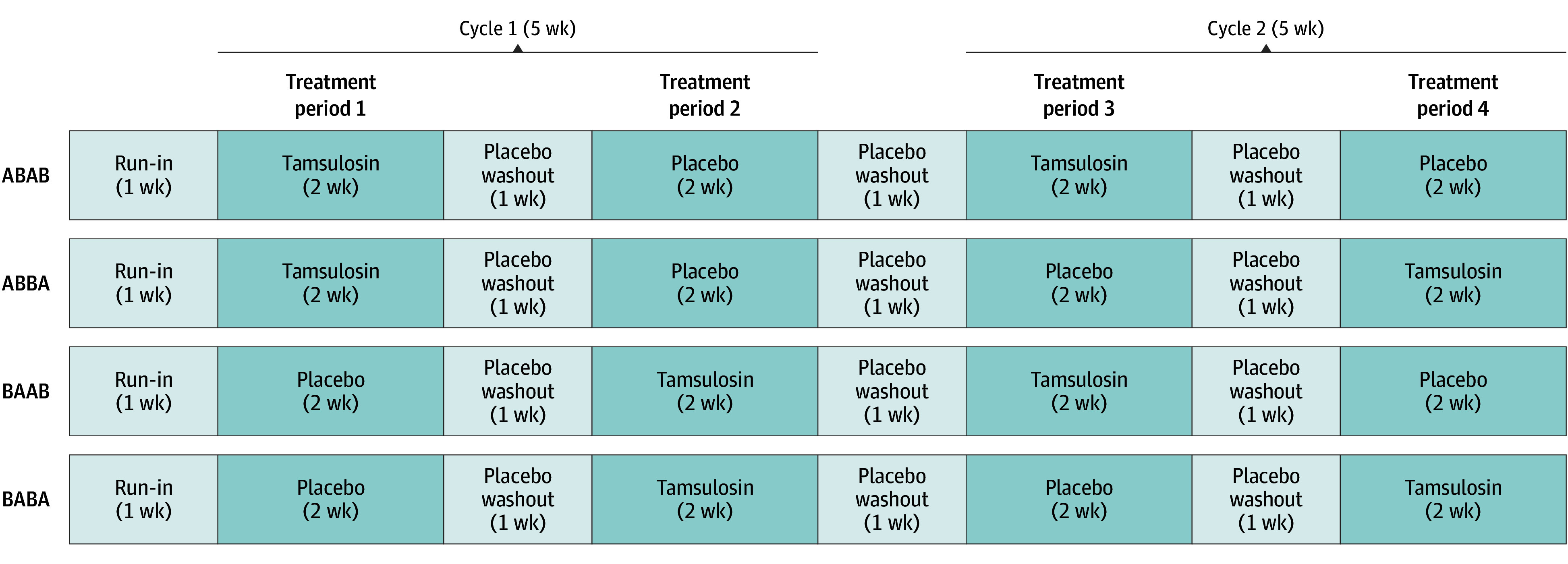
Study Design Chart Including All 4 Possible Crossover Combinations

This was a 12-week double-blind, placebo-controlled, multiple crossover randomized clinical trial recruiting participants from urology clinics within the University of California, San Francisco (UCSF) Health system between September 2021 and July 2022. Based on the published pharmacokinetics (half-life of 14 to 15 hours; steady state by the 5th day), pharmacodynamics and expected time frame of symptomatic relief from tamsulosin (1-2 weeks),^23^ we decided on a 1-week placebo-blinded washout period. We also conducted a confirmatory pharmacokinetic validation substudy among 14 older men who met our N-of-1 study inclusion and exclusion criteria, which confirmed that the washout period was more than 14 elimination half-lives based on the mean in this study population and more than 6 elimination half-lives based on the greatest observed half-life in the substudy (eMethods and eFigure 2 in Supplement 1). The protocol included a 1-week placebo run-in followed by two 5-week cycles. Each 5-week cycle included two 2-week treatment periods (tamsulosin and placebo) in randomized AB or BA sequence, separated by the washout period (Figure 1). At the end of each participants’ N-of-1 trial, they were provided a graphical representation of their daily symptom survey responses and offered a phone call with a study investigator (S.R.B.) to review their results (eFigure 2 in Supplement 1). All participants provided signed informed consent and the study was approved by the UCSF Human Subjects Committee and institutional review board. The study followed the Consolidated Standards of Reporting Trials extension for reporting N-of-1 trials (CONSORT-CENT) guidelines and the full trial protocol is included in Supplement 2.

### Participants, Recruitment, and Enrollment

**Figure 2.**
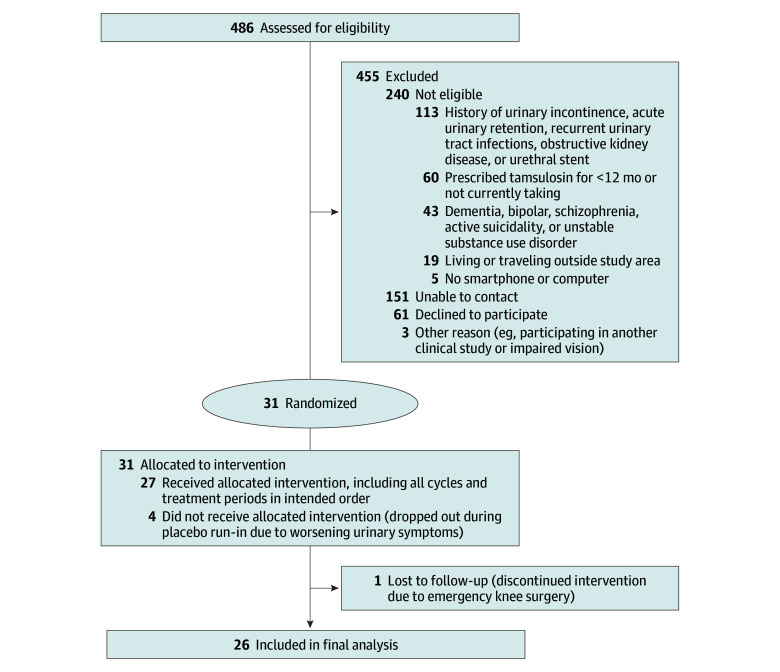
Study Flow Diagram

We identified all male patients aged 55 to 80 years who were followed-up by a urologist at UCSF Health, had an *International Statistical Classification of Diseases and Related Health Problems, Tenth Revision* diagnosis consistent with BPH and an active prescription for tamsulosin, were able to speak and read in English, and agreed to be contacted about research studies via the electronic health record (486 individuals). Study staff used electronic health record data to determine initial eligibility and completed a screening call to finalize eligibility. We excluded men based on the following criteria: American Urological Association Symptom Index (AUASI) less than 5 or greater than 25; history of urinary incontinence, urinary retention, urinary tract infections, obstructive kidney disease, or urethral stent; prescribed tamsulosin for less than 12 months or not currently taking tamsulosin; or other barriers to completing the protocol (Figure 2).

### Randomization and Allocation

We used block randomization to assign each participant to 2 cycles, with each cycle containing 1 period of daily tamsulosin and 1 period of daily placebo in random order, using a randomization schedule created by a study data analyst who was blinded to the period definition (K.L.). Allocation was applied via sequentially numbered prefilled bubble packs labeled only by number of days in the study. The order of treatment periods within a cycle were random (eg, ABAB, BABA, ABBA, or BAAB) and participants were not informed of the order or duration of the treatment periods or washout periods.

### Intervention

The N-of-1 design was identical for all participants, with the exception of the tamsulosin dose, which was selected based on the dose (0.4 mg or 0.8 mg) they were previously receiving as chronic tamsulosin therapy. To ensure that the study drugs appeared identical, tamsulosin was re-encapsulated to match the placebo pill. Study drugs were mailed to participants in bubble packs as described previously.

### Outcomes

LUTS were assessed daily using a modified version of the 7-item AUASI^24^ with a 24-hour recall (eMethods in Supplement 1). Total scores range from 0 to 35. The minimal clinically important difference (MCID) in the AUASI with a recall period of 1 month is between 2 and 6 points depending on the study and baseline AUASI score, although the MCID has not been established for the 24-hour recall modified version.^25,26^ The standard AUASI with a 1-month recall was collected at baseline and used to calculate clinically relevant categories of LUTS severity at baseline: 0 to 7 (none or mild), 8 to 19 (moderate), and 20 to 35 (severe).^27^ Baseline LUTS treatment was assessed, including tamsulosin dose and concurrent 5α-reductase inhibitor use. We also report several feasibility outcomes (recruitment and retention rates, run-in tolerability, adherence to daily assessments, and multiple crossover periods).

### Safety and Adverse Drug Events

The 12 most common tamsulosin adverse effects were assessed daily by asking participants whether they had experienced any of the symptoms in the past 24 hours, including dizziness, headache, erectile dysfunction, decreased libido, rhinitis, fatigue, insomnia, diarrhea, constipation, nausea, back pain, and other. Response options for each individual symptom were “not at all bothered,” “somewhat bothered,” “very bothered,” and “extremely bothered.”(range 0-3). To quantify the total tamsulosin adverse effect burden, a daily summary adverse effect score (range 0-36) was calculated by summing bother scores reported for all symptoms. Participants were also provided study staff contact information for any additional adverse event reporting during the study.

### Other Measurements

Demographics were collected at baseline, including age, marital status, education, and self-reported race and ethnicity. The total number of 11 chronic diseases and LUTS-related health conditions was quantified as a sum. Social determinants of health were assessed using the IOM tool.^28^ Health-related quality of life was assessed using the Patient-Reported Outcomes Measurement Information System 29 v2.0 Profile.^29^ Tamsulosin nonadherence was assessed using the Voils Dose-Nonadherence Scale.^30,31^ Attitudes toward deprescribing medications (not specifically tamsulosin) were assessed using the Revised Patients’ Attitudes Toward Deprescribing (rPATD) instrument.^32^ Additional details on covariate measurement, including perceived benefit of and satisfaction with chronic tamsulosin therapy, are provided in the eMethods in Supplement 1.

### Statistical Analysis

Normally distributed continuous variables were described as mean (SD) and compared via *t *tests or analysis of variance. Categorical variables were compared using χ^2^ tests. The primary outcome for the N-of-1 trials was daily AUASI score. Data from all washout periods were excluded. To estimate variation in daily AUASI score and daily summary adverse effect score, we used multivariable adjusted linear mixed models with individual-specific intercepts and treatment effects and an unstructured variance-covariance matrix. Treatment, day, and period were included as independent variables. We used the individual-specific effect estimates (estimated best linear unbiased predictions^33^) of tamsulosin treatment on daily AUASI and their standard errors to define strong responders (upper bound of 95% CI ≤−6.0), moderate responders (upper bound of 95% CI ≤0.0 and >-6.0), and minimal or nonresponders (upper bound of 95% CI ≥0) eFigure 3 in Supplement 1). These groups were created based on upper bounds of the 95% CI since it represents a conservative estimate of efficacy (eg, smallest benefit) for an individual who might be considering deprescribing, although we recognize that the MCID for the 24-hour recall period has not been established. Given the clinical relevance of failing to tolerate the placebo run-in, we include these participants whenever summarizing the responder groups. To visualize individual treatment effects among those who were able to tolerate the N-of-1 protocol, we created a bar graph with the mean effect of tamsulosin on daily AUASI and 95% CI for each participant. To visualize difference in mean treatment effect by baseline LUTS severity, we generated a plot of the mean daily AUASI score during tamsulosin and placebo periods for each participant. Sensitivity analyses are described in the eMethods in Supplement 1.

We determined our sample size of this based on expected attrition rates. Based on our previously published protocol study and prior mobile health studies,^34,35^ we expected to fail to meet our study goals (ie, achieve sufficient daily questionnaire and N-of-1 trial completion rates to determine the efficacy of tamsulosin compared with placebo for each study participant) at least 10% of the time. Therefore, with a sample size of 20 participants, we have 90% power to observe at least 1 failure during this study.^36,37^ All analyses were performed using SAS version 9.4 (SAS Institute). *P* < .05 was considered statistically significant for all tests. Data were analyzed from November 2022 to October 2024.

## Results

Out of 486 individuals screened for eligibility, 31 were enrolled. The mean (SD) age of participants was 68.5 (5.9) years; 2 participants (6.5%) were Black, 29 (93.5%) were non-Hispanic, and 27 (87.1%) were White. Baseline characteristics are listed in Table 1. Most participants had severe LUTS at baseline; the mean (SD) AUASI score was 20.0 (5.9) and the mean (SD) bother score was 3.0 (1.5).

**Table 1.** Characteristics of 31 Enrolled Study Participants

Characteristic	Participants, No. (%)
Demographics	
Age, mean (SD), y	68.5 (5.9)
Married	17 (54.8)
College education	8 (25.8)
Self-reported race	
Asian	1 (3.2)
Black	2 (6.5)
White	27 (87.1)
Other^a^	1 (3.2)
Hispanic, Latino, or Spanish	2 (6.5)
Ability to pay for basic living expenses	
Not difficult at all	25 (80.6)
Somewhat or very difficult	5 (16.1)
Prefer not to answer	1 (3.2)
Health-related behaviors	
Physical activity^b^	
Inactive	6 (19.4)
Insufficiently active	8 (25.8)
Sufficiently active	16 (51.6)
Unknown or missing	1 (3.2)
Current smoking	0
Heavy alcohol use^c^	8 (25.8)
Self-reported comorbidities	
Diabetes	7 (22.6)
Hypertension	15 (51.7)
Coronary artery disease	7 (22.6)
Congestive heart failure	1 (3.2)
Chronic obstructive pulmonary disease	2 (6.5)
No. of comorbidities, mean (SD)	1.7 (1.8)
PROMIS 29 v2.0, mean (SD)	
Physical health summary T-score	50.9 (8.3)
Mental health summary T-score	51.6 (6.6)

Abbreviation: PROMIS, Patient-Reported Outcomes Measurement Information System.

^a^ The participant who selected this response did not provide additional information regarding their race.

^b^ Calculated as minutes per week engaged in moderate to strenuous activity and was categorized as follows: inactive, 0 minutes per week; insufficiently active, 1 to 149 minutes per week; sufficiently active, 150 or more minutes per week.

^c^ Alcohol use was tabulated as a composite value integrating alcohol consumption frequency (“How often do you have a drink?”) and density (“How many standard drinks on a typical day?” and “How often do you have ≥6 drinks on one occasion?”) and a score of 4 or higher indicated a positive screen for heavy alcohol use.

**Figure 3.**
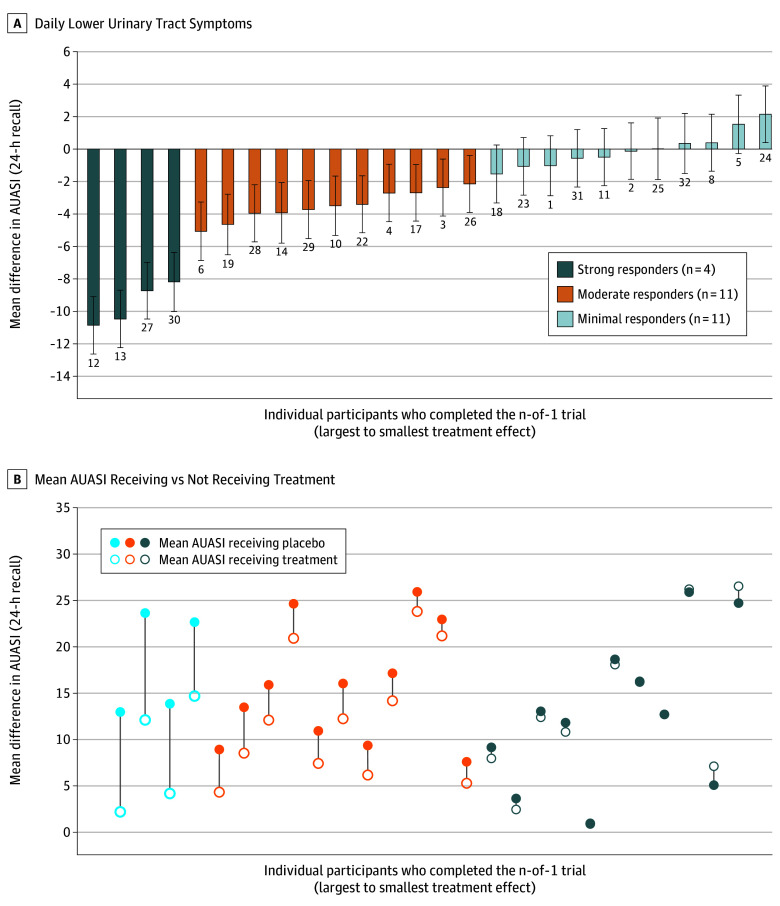
Bar Chart Showing Individual-Level Treatment Effects and 95% CIs of Tamsulosin vs Placebo Individual level estimates of mean treatment difference and 95% CIs were calculated using linear mixed models adjusted for period and time. Participant study IDs are included below each individual estimate in panel A and ordered by participant with the largest to smallest treatment effect. AUASI indicates American Urological Association Symptom Index.

Of the 31 enrolled participants, 4 did not tolerate the placebo run-in period and dropped out due to worsening LUTS, 1 participant dropped out during the first cycle due to an emergency knee surgery unrelated to the treatment intervention, and 26 participants completed the full N-of-1 protocol. Participants completing the N-of-1 protocol reported a mean (SD) of 53.5 (2.8) total daily AUASI assessments. Individual-level effects of tamsulosin treatment on daily LUTS severity are shown in Figure 3A, which demonstrate that 11 (36.7%) were minimal or nonresponders, 11 (36.7%) moderate responders, 4 (13.3%) strong responders, and 4 (13.3%) did not tolerate the 1-week placebo 4 run-in due to worsening symptoms. Individual-level treatments effects did not vary by treatment sequence (*F*_3_ = 1.52; *P* = .21) or by baseline AUASI score (*t*_24_ = −0.79; *P* = .43) (Figure 3B). The range of individual mean differences in 24-hour recall AUASI score with tamsulosin vs placebo was −10.9 (95% CI, −12.6 to −9.1) to 2.1 (95% CI, 0.4 to 3.9), and the overall group-level mean difference was −2.96 (95% CI, −4.37 to −1.54) (eTable 1 in Supplement 1). Adverse events during the N-of-1 protocol were common: 24 out of 26 participants (92.3%) reported at least 1 day with a possible adverse drug reaction and the mean (SD) number of days with a possible adverse drug reaction was 34 (18) days. While most participants experienced similar adverse effects while talking tamsulosin or placebo, the severity of possible adverse drug reactions were statistically greater for 2 (18.2%) of tamsulosin minimal or nonresponders, 2 (18.2%) of moderate responders, and 0 strong responders (eTable 1 in Supplement 1).

Table 2 includes baseline measures of perceived benefit from and satisfaction with chronic tamsulosin therapy, adherence, and deprescribing attitudes by responder status. At baseline, 7 minimal or nonresponders (63.6%), 9 moderate responders (81.8%), 1 strong responder (25.0%), and 3 who failed the placebo run-in (75.0%) perceived receiving “much” benefit from tamsulosin and almost all were at least a “little satisfied” with chronic tamsulosin therapy. Minimal or nonresponders and those who failed placebo run-in were more likely to report tamsulosin nonadherence compared with moderate and strong responders. Based on rPATD scores, overall medication burden was low and involvement in medications was high among all participants, although minimal or nonresponders and those who failed placebo run-in reported a greater willingness to stop their medications compared with strong responders. Additional baseline characteristics stratified by responder status are listed in eTable 2 in Supplement 1.

**Table 2.** Baseline Tamsulosin Satisfaction, Adherence, and Attitudes Toward Tamsulosin Deprescribing Among 30 Study Participants Who Attempted the Full Protocol, Stratified by Tamsulosin Treatment Response^a^

Variable	Participants, No. (%)				*P* value^b^
	Tamsulosin treatment response			Failed run-in (n = 4)	
	Minimal or none (n = 11)	Moderate (n = 11)	Strong (n = 4)		
Tamsulosin satisfaction and adherence					
Perceived benefit from tamsulosin					
None	0	1 (9.1)	0	0	.26
Little	4 (36.4)	1 (9.1)	3 (75.0)	1 (25.0)	
Much	7 (63.6)	9 (81.8)	1 (25.0)	3 (75.0)	
Satisfaction with chronic tamsulosin therapy					
Much dissatisfied	0	0	1 (25.0)	0	.21
Little dissatisfied	2 (18.2)	1 (10.0)	0	0	
Little satisfied	2 (18.2)	2 (20.0)	2 (50.0)	0	
Much satisfied	7 (63.6)	7 (70.0)	1 (25.0)	4 (100.0)	
Tamsulosin nonadherence^c^	4 (36.4)	1 (9.1)	0	3 (75.0)	.04
Most common reasons for tamsulosin nonadherence					
I was afraid it may affect my sexual performance	2 (18.2)	3 (27.3)	0	1 (25.0)	.58
I worried about taking it for the rest of my life	3 (27.3)	1 (9.1)	0	0	.44
I felt I did not need it	2 (18.2)	1 (9.1)	0	0	.72
I ran out of medication	3 (27.3)	0	0	1 (25.0)	.13
Deprescribing					
rPATD subscores, mean (SD)^d^					
Burden	2.6 (0.7)	2.4 (1.0)	2.2 (1.0)	2.5 (0.4)	.92
Appropriateness	2.6 (0.6)	2.4 (0.5)	2.3 (0.9)	2.6 (0.4)	.76
Willingness to stop medications	2.4 (0.5)	2.1 (0.7)	1.6 (0.4)	2.6 (0.4)	.04
Involvement in medications	4.2 (0.7)	4.3 (0.5)	4.6 (0.5)	4.0 (0.3)	.39

Abbreviation: rPATD, Revised Patients’ Attitudes Toward Deprescribing.

^a^ Includes 4 individuals who attempted the n-of-1 protocol and dropped out during the 1-week placebo run-in due to worsening symptoms.

^b^ *P* value calculated using analysis of variance for continuous variables and χ^2^ tests for categorical variables.

^c^ Tamsulosin nonadherence based on the DOSE-Nonadherence scale and defined as any response other than “none of the time” to “I missed my medicine,” “I skipped a dose of medicine,” and “I did not take a dose of my medicine.”

^d^ Higher rPATD subscores correspond to a greater perceived burden, concerns about stopping, and involvement. Questions in the appropriateness subscore were reverse scored so that a higher score represents a greater belief in appropriateness.

In the confirmatory pharmacokinetic validation substudy, we confirmed that the half-life of tamsulosin in this study population was 11.81 hours (eFigure 1 in Supplement 1). In sensitivity analyses, excluding the first 7 days of each treatment period did not materially change the results. In exploratory analyses, we evaluated change in quality of life, global urinary bother, and perceived benefit and satisfaction with tamsulosin from baseline to after completion of the N-of-1 protocol (eTable 3 in Supplement 1). Fewer study participants reported perceived benefit or satisfaction with tamsulosin after completing the N-of-1 protocol, although differences were not statistically significant.

## Discussion

In this series of placebo-controlled N-of-1 deprescribing trials, we observed significant heterogeneity of response to tamsulosin deprescribing among older men receiving chronic tamsulosin therapy for BPH. Of 30 participants who completed the full N-of-1 protocol, 4 did not tolerate the placebo run-in due to worsening symptoms, 4 demonstrated a strong response to tamsulosin, and the remaining participants were evenly split between minimal or nonresponders and moderate responders. While the overall group-level mean difference in 24-hour recall AUASI (predicted mean difference, −2.96) approximated previously published tamsulosin RCTs (pooled mean difference, −2.13),^38^ the individual-level response to tamsulosin was heterogeneous (predicted mean difference, −10.9 to 2.1). We also found that common tamsulosin-related adverse drug events were highly prevalent but they occurred at similar frequencies while talking tamsulosin and placebo for most study participants, suggesting that participants were reporting symptoms that were unrelated to tamsulosin due to misattribution of background symptoms or nocebo effects^39^ (when placebos produce adverse effects that arise from negative expectations about a treatment). Although potential inferences were limited by the small sample size, tamsulosin treatment response did not appear to be associated with perceived benefit from and satisfaction with chronic tamsulosin therapy, whereas minimal or nonresponders were somewhat less adherent and more willing to stop their medications. The results of this proof-of-concept study suggest that placebo-controlled N-of-1 deprescribing trials can be used to quantify the individualized benefits and harms of chronic tamsulosin therapy for older men with lower urinary tract symptoms.

We are not aware of any prior N-of-1 trials for nonmalignant urologic conditions, including LUTS. However, in this small but rigorous series of double-blind, placebo-controlled N-of-1 deprescribing trials, we demonstrated that this study design can be used to define tamsulosin treatment effects for older men receiving chronic tamsulosin therapy. There are several clinical implications of this study. First, we observed highly heterogeneous tamsulosin treatment effects across a relatively homogeneous study population with similar demographics, clinical characteristics, chronic tamsulosin use, and management by clinical experts in BPH. These findings suggest that it may be inappropriate to counsel individual older men to expect previously reported group-level tamsulosin effects and a much larger range of possible effectiveness should be communicated clearly. Although the sample was small, the overall group-level effect estimates matched prior tamsulosin RCTs, suggesting that these results are likely generalizable to the target population of those RCTs, although heterogeneity of response to tamsulosin may be even greater in the primary care setting compared with urology. Second, 1 in 3 participants had minimal or no effect of tamsulosin vs placebo on LUTS severity and, therefore, may be excellent candidates for tamsulosin deprescribing, which is important knowledge for both patients and clinicians. These results support the use of discrete medication trials rather than assuming lifelong tamsulosin treatment for older men with LUTS due to suspected BPH, and suggest a need for tamsulosin deprescribing to be addressed in clinical guidelines. Third, baseline LUTS severity on placebo was not associated with tamsulosin treatment effect, suggesting that empiric trials with close follow-up and monitoring for responsiveness are necessary to determine whether tamsulosin should be continued. Although we were not adequately powered to evaluate predictors of individual-level tamsulosin treatment effect, the N-of-1 study design is ideal for addressing this research question and, as an example, we visualized the association between baseline AUASI and treatment response. A larger series of N-of-1 trials should be conducted to determine the efficacy of tamsulosin in vulnerable populations that are underrepresented in traditional BPH trials and to identify predictors of tamsulosin treatment response.

Another important finding of this study is the high degree of perceived benefit and satisfaction with chronic tamsulosin therapy, including older men who were later determined to have minimal or no effect of tamsulosin. The large placebo effect has been well-established in LUTS medication and sham surgical trials, and is a known barrier to accurately identifying tamsulosin responders and nonresponders.^38,40,41^ Since they have continued chronic tamsulosin therapy under the supervision of a urologist, we expected our study population to perceive a net benefit and to report high satisfaction with tamsulosin. However, since minimal or nonresponders also reported high perceived benefit and satisfaction, prescribing clinicians need to carefully balance possible harms against an apparent placebo effect. Conversely, minimal or nonresponders did have a higher prevalence of tamsulosin nonadherence and willingness to stop their medications compared with strong responders, suggesting that they were not entirely satisfied. These differences provide early evidence that there is a potentially large population of older men receiving chronic tamsulosin therapy that would benefit from deprescribing.

N-of-1 trials have been previously recognized as a useful tool for implementing evidence-based and personalized medicine in settings where the target study population is small (eg, rare diseases^42,43,44,45^), heterogenous (eg, geriatrics^46,47,48^), or underrepresented in traditional clinical trials for any reason. As recently illustrated by Goyal et al,^46^ N-of-1 trials may be particularly useful for evidence-based deprescribing due to their ability to generate medication response data, allay patient and clinician concerns about deprescribing, and, in some cases, circumvent the time constraints of clinical encounters. Although we used double-blind RCTs for this N-of-1 series, unblinded N-of-1 studies can still be effective^49^ and more accurately reflect clinical decision-making.^46^ Taken together, our study is an example of how N-of-1 trials can be used to accurately test treatment efficacy for common diseases in vulnerable and underrepresented populations.

### Strengths and Limitations

Strengths of this study include rigorous blinding and use of a placebo, daily outcome assessment to maximize precision of individual-level effect estimates, and validation of key study design assumptions, including delayed treatment effects and tamsulosin half-life.

This study also has several limitations. First, we only assessed a single treatment period length (2-weeks) and washout period (1-week), although these period lengths were supported by our pharmacokinetics substudy and sensitivity testing. Second, we did not have urodynamics, uroflowmetry, prostate volume, or postvoid residual since we focused on the clinically relevant patient-reported outcome of LUTS severity, which limits mechanistic phenotyping and exploration of these measures as predictors of treatment response. Third, it remains unknown how generalizable these findings are to alternative clinical settings and more racially and/or ethnically diverse, frail, or cognitive impaired men. Furthermore, men willing to participate in a deprescribing trial may respond differently than men in the general population. Fourth, the MCID of the modified version of the AUASI with a 24-hour recall has not been established and will certainly vary by individual, therefore the grouping of treatment response should be used to guide interpretation of the results but not to definitively classify individuals. Additionally, our sample size was too small to test predictors of tamsulosin treatment response or BPH complications and other long-term adverse effects of N-of-1-guided deprescribing.

## Conclusions

In this randomized N-of-1 crossover clinical trial of tamsulosin deprescribing, we demonstrated that this study design can precisely quantify the individualized benefits and harms of chronic tamsulosin therapy for older men with lower urinary tract symptoms. Specifically, we demonstrated significant heterogeneity in treatment response and identified a significant proportion of participants that may benefit from discontinuing tamsulosin. This study highlights the need to counsel patients on the heterogeneity of tamsulosin treatment effects and supports the clinical use of empiric tamsulosin trials rather than lifelong therapy. Larger studies are needed to confirm generalizability across clinical settings and patient populations, to identify predictors of tamsulosin response, and to test the effect of N-of-1-guided deprescribing on clinical outcomes.

## Notes

Supplement 1. eMethods. eTable 1. Group-Level and Individual-Level Effects of Tamsulosin Versus Placebo on Daily Lower Urinary Tract Symptoms Severity and Side Effects Estimated Using Linear Mixed Models eTable 2. General Characteristics of 30 Study Participants Who Attempted the Full Protocol, Stratified by Tamsulosin Treatment Response eTable 3. Change in Quality of Life, Urinary Bother, and Tamsulosin Nonadherence From Baseline to Follow-Up After Competition of the N-of-1 Protocol Among the 26 Protocol Completers eFigure 1. Categories of Tamsulosin Treatment Response Based on the Upper Bound of 95% CIs for Individual-Specific Effect Estimates eFigure 2. Pharmacokinetics of Tamsulosin Elimination in Older Men Receiving Chronic Tamsulosin Therapy eFigure 3. Example of a Report Provided to a Study Participant With a Strong Tamsulosin Response After Completing the N-of-1 Protocol eReferences.

Supplement 2. Trial Protocol

Supplement 3. Data Sharing Statement
