# Supplementary material for: Tamsulosin Deprescribing for Lower Urinary Tract Symptoms in Older Men: A Randomized Clinical Trial
Source: JAMA Netw Open. 2026 Jul 6;9(7):e2621639. doi: 10.1001/jamanetworkopen.2026.21639 (PMC13338806; doi:10.1001/jamanetworkopen.2026.21639)
Supplement: Supplement 1. — eMethods. eTable 1. Group-Level and Individual-Level Effects of Tamsulosin Versus Placebo on Daily Lower Urinary Tract Symptoms Severity and Side Effects Estimated Using Linear Mixed Models eTable 2. General Characteristics of 30 Study Participants Who Attempted the Full Protocol, Stratified by Tamsulosin Treatment Response eTable 3. Change in Quality of Life, Urinary Bother, and Tamsulosin Nonadherence From Baseline to Follow-Up After Competition of the N-of-1 Protocol Among the 26 Protocol Completers eFigure 1. Categories of Tamsulosin Treatment Response Based on the Upper Bound of 95% CIs for Individual-Specific Effect Estimates eFigure 2. Pharmacokinetics of Tamsulosin Elimination in Older Men Receiving Chronic Tamsulosin Therapy eFigure 3. Example of a Report Provided to a Study Participant With a Strong Tamsulosin Response After Completing the N-of-1 Protocol eReferences. [file jamanetwopen-e2621639-s001.pdf]

Supplemental Online Content

Bauer SR, Kenfield SA, Oni-Orisan A, et al. Tamsulosin deprescribing for lower urinary tract symptoms in older men. *JAMA Netw Open*. 2026;9(7):e2621639. doi:10.1001/jamanetworkopen.2026.21639

eMethods.
eTable 1. Group-Level and Individual-Level Effects of Tamsulosin Versus Placebo on Daily Lower Urinary Tract Symptoms Severity and Side Effects Estimated Using Linear Mixed Models
eTable 2. General Characteristics of 30 Study Participants Who Attempted the Full Protocol, Stratified by Tamsulosin Treatment Response
eTable 3. Change in Quality of Life, Urinary Bother, and Tamsulosin Nonadherence From Baseline to Follow-Up After Competition of the N-of-1 Protocol Among the 26 Protocol Completers
eFigure 1. Categories of Tamsulosin Treatment Response Based on the Upper Bound of 95% CIs for Individual-Specific Effect Estimates
eFigure 2. Pharmacokinetics of Tamsulosin Elimination in Older Men Receiving Chronic Tamsulosin Therapy
eFigure 3. Example of a Report Provided to a Study Participant With a Strong Tamsulosin Response After Completing the N-of-1 Protocol
eReferences.

This supplemental material has been provided by the authors to give readers additional information about their work.

eMethods

Additional Study Procedures

The random allocation sequence generated by a blinded data analyst included both cycle and period sequence. The personnel who mailed participants the bubble packs did not have access to the random allocation sequence. The data analyst, principal investigators, and participants remained fully blinded. With the exception of their tamsulosin prescription, participants received usual care during their n-of-1. There were no changes to the trial design after it commenced. There was no patient or public involvement in the design, conduct or reporting of this trial. Participants were asked to complete daily symptom questionnaires. If a participant did not complete the daily symptom questionnaire for more than 3 consecutive days, they were contacted by study staff.

Outcomes

LUTS were assessed daily using a modified version of the 7-item American Urological Association Symptom Index (AUASI)[1], which assesses urinary frequency, urgency, intermittency, straining, weak urinary stream, incomplete bladder emptying, and nocturia. Specifically, we used a previously adapted version of the AUASI with a recall period of 24 hours (versus 1 month) to allow for more frequent assessments during N-of-1 trials.[2,3] For example, to evaluate urinary urgency, participants were asked “In the past 24 hours, how often have you found it difficult to postpone urination?” Response options included “Not at all”, “Less than 1 time in 5”, “Less than half the time”, “About half the time”, “More than half the time”, or “Almost always”. Responses to each item are on an ordinal scale with values ranging from 0 to 5 (higher = more frequent symptoms).

The AUASI was collected at baseline and used to calculate clinically relevant categories of LUTS severity at baseline: 0 to 7 (none/mild), 8 to 19 (moderate), and 20 to 35 (severe).[4] We also calculated validated AUASI subscores separately for storage symptoms (urgency, frequency, nocturia) and for voiding symptoms (incomplete emptying, intermittency, weak stream, straining).[5] Urinary bother was assessed at baseline using the AUASI global bother question[1,6] and at baseline and follow-up using Lower Urinary Tract Research Network 29-item Symptom Index (LURN SI-29) global bother question.[7] Baseline LUTS treatment, including tamsulosin dose and concurrent 5α-reductase inhibitor use, were assessed.

Other Measurements

Demographics were collected at baseline, including age, marital status, level of educational attainment, and race/ethnicity. Self-reported health conditions were assessed and total number of comorbidities was quantified as a sum of the following chronic diseases and LUTS-related health conditions: hypertension, heart disease, angina, heart failure, chronic obstructive pulmonary disease, diabetes, stroke, Parkinson's, visual impairment, and prostatitis. We evaluated the participants ability to pay for basic living expenses (not hard at all, somewhat or very hard, or prefer not to answer).[8] Physical activity was calculated as minutes per week engaged in moderate to strenuous activity and was categorized as inactive (0 min/week), insufficiently active (1-149 min/week), and sufficiently active (150+ min/week) according to Institute of Medicine clinical guidelines.[8] Other health-related behaviors, including smoking and alcohol intake, were also assessed. Health-related quality of life was assessed using the PROMIS 29 v2.0 Profile and validated physical and mental health summary scores were reported separately.[9] Perceived benefit of chronic tamsulosin therapy was assessed with the question "Have you had any benefit from your tamsulosin medication?" and response options included: No, Yes – little benefit, and Yes – much benefit. Satisfaction with chronic tamsulosin therapy was assessed with the question "Taking all things into account, are you satisfied with your tamsulosin medication?" and response options included: No, Yes – a little satisfied, and Yes – very satisfied. We assessed self-reported tamsulosin non-adherence (both extent of and reasons for non-adherence) at baseline and follow-up using the Voils Dose-Nonadherence Scale – permission was granted by Duke University to use this instrument.[10,11] Because few study participants reported tamsulosin nonadherence and the distribution of the Dose-Nonadherence Scale was skewed, a binary variable was created to categorize patients as adherent (*none of the time* to all three items) or nonadherent (any response other than *none of the time* to any item) when asked "I missed my medicine," "I skipped a dose of medicine," and "I did not take a dose of my medicine." Attitudes towards deprescribing medications (not specifically tamsulosin) were assessed at baseline using the Revised Patients' Attitudes Towards Deprescribing (rPATD) instrument.[12]

Pharmacodynamics sub-study

Treatment and washout periods chosen based on previously published pharmacokinetic/pharmacodynamic data[13] were confirmed through a pharmacokinetics substudy in 14 distinct participants selected with the same inclusion and exclusion criteria as the parent n-of-1 trial but who enrolled after the target sample size had been reached. Following ≥7 daily doses of 0.8mg tamsulosin (to reach steady state), participants discontinued therapy and underwent blood draws at 24 (trough, C_{24}), 48 (C_{48}), and 72 hours (C_{72}) post-dose. Samples were processed, aliquoted, and stored at -80 degrees Celsius for future quantification.

Total plasma tamsulosin was measured using a Sciex Triple Quad 6500+ tandem mass spectrometer coupled with waters ultra-performance liquid chromatography. Briefly, each plasma sample (20 microliters) was mixed with 20 microliters internal standard and 80 microliters acetonitrile for protein precipitation. Following centrifugation, 40 microliters of supernatant was mixed with 120 microliters of water. Subsequently, 5 microliters of the resulting solution were injected into the liquid chromatography tandem mass spectrometer for quantification. Based on reported tamsulosin pharmacokinetic parameters, we set a target calibration range of 0.1 to 20 nanograms per milliliter.

To generate tamsulosin elimination half-life values ($T_{1/2}$) for each participant, we used elimination rate constants (k_e, based on evidence of first-order elimination kinetics for tamsulosin [PMID: 20642551]) in the following formula:

$$T_{1/2} = 0.693/k_e$$

The k_e constant was estimated as the slope of the fitted line for each natural log-transformed concentration-time profile created from the C_{24}, C_{48}, and C_{72} data points.

Data Analysis

The model incorporated individual-specific intercepts and treatment effects allowing estimation of individual-specific response to treatment for both AUASI score (shown below) and daily summary side effect score:

$$AUASI_{ij} = \beta_1 + \beta_2 Treatment_{ij} + \beta_3 Day_{ij} + \beta_4 Period_{ij} + b_{i1} + b_{i2} Treatment_{ij} + \varepsilon_{ij}$$

To determine if there were carryover effects, we first included a variable indicating the sequence of each period (tamsulosin then placebo or placebo then tamsulosin) in the linear mixed model. Since this variable was not statistically significant, we dropped it from the model and conducted a sensitivity

analysis excluding the first 7 days of each treatment period. To evaluate whether treatment effect was correlated with AUASI score on placebo, we calculated the correlation between the random intercept and random treatment effect and used a likelihood ratio test to compare models with an unstructured covariance matrix versus an independent covariance matrix. Outcome data was assumed missing at random.

eTable 1. Group-level and individual-level effects of tamsulosin versus placebo on daily lower urinary tract symptoms severity and side effects estimated using linear mixed models.

	**AUASI (24-hour recall)**		**Side Effect Summary Score**	
	**Mean Difference* (95% CI)**	**P-value**	**Mean Difference* (95% CI)**	**P-value**
Group-level	-2.96 (-4.37,-1.54)	<0.001	0.005 (-0.242, 0.252)	0.97
Individual-level				
Participant 12	-10.86 (-12.63, -9.09)	<0.001	-0.45 (-0.93, 0.03)	0.07
Participant 13	-10.47 (-12.24, -8.70)	<0.001	0.01 (-0.47, 0.49)	0.97
Participant 27	-8.73 (-10.47, -6.99)	<0.001	0.05 (-0.42, 0.53)	0.82
Participant 30	-8.19 (-10.01, -6.37)	<0.001	-0.32 (-0.81, 0.17)	0.20
Participant 6	-5.07 (-6.87, -3.27)	<0.001	-0.16 (-0.65, 0.33)	0.52
Participant 19	-4.65 (-6.51, -2.78)	<0.001	0.12 (-0.38, 0.62)	0.63
Participant 28	-3.96 (-5.72, -2.20)	<0.001	-0.12 (-0.60, 0.36)	0.63
Participant 14	-3.93 (-5.80, -2.07)	<0.001	-0.40 (-0.90, 0.10)	0.12
Participant 29	-3.73 (-5.52, -1.95)	<0.001	-0.13 (-0.62, 0.36)	0.60
Participant 10	-3.50 (-5.33, -1.67)	<0.001	0.44 (-0.05, 0.94)	0.08
Participant 22	-3.41 (-5.16, -1.65)	<0.001	-0.48 (-0.96, 0.00)	0.05
Participant 4	-2.71 (-4.48, -0.94)	0.003	0.01 (-0.47, 0.49)	0.96
Participant 17	-2.70 (-4.44, -0.95)	0.002	-0.09 (-0.57, 0.38)	0.70
Participant 3	-2.37 (-4.13, -0.62)	0.008	-1.03 (-1.51, -0.55)	<.0001
Participant 26	-2.15 (-3.91, -0.39)	0.02	-0.28 (-0.76, 0.20)	0.26
Participant 18	-1.54 (-3.32, 0.25)	0.09	0.17 (-0.32, 0.65)	0.50
Participant 23	-1.07 (-2.84, 0.70)	0.24	0.11 (-0.37, 0.59)	0.65
Participant 1	-1.03 (-2.88, 0.82)	0.28	-0.18 (-0.68, 0.32)	0.48
Participant 31	-0.57 (-2.34, 1.20)	0.53	-0.15 (-0.64, 0.33)	0.54
Participant 11	-0.50 (-2.26, 1.26)	0.58	-0.39 (-0.87, 0.09)	0.11
Participant 2	-0.13 (-1.87, 1.61)	0.88	1.64 (1.16, 2.12)	<.0001
Participant 25	0.01 (-1.89, 1.91)	0.99	0.50 (-0.01, 1.01)	0.06
Participant 32	0.34 (-1.51, 2.19)	0.72	0.20 (-0.30, 0.70)	0.44
Participant 8	0.39 (-1.37, 2.15)	0.67	1.18 (0.70, 1.66)	<.0001
Participant 5	1.52 (-0.28, 3.32)	0.10	-0.26 (-0.75, 0.22)	0.29
Participant 24	2.14 (0.40, 3.88)	0.02	0.15 (-0.33, 0.63)	0.54

* Group-level and individual level estimates of mean treatment difference, 95% confidence intervals, and p-values were calculated using linear mixed models adjusted for period and time. P-value based on the null hypothesis of no treatment effect for each individual.

eTable 2. General characteristics of 30 study participants who attempted the full protocol*, stratified by tamsulosin treatment response.

Variable n (%)	Tamsulosin Treatment Response				P-value‖
	Minimal/ None (n=11)	Moderate (n=11)	Strong (n=4)	Failed Run-in (n=4)	
**Demographics**					
Age, years, mean (SD)	70.8 (5.2)	66.5 (6.4)	69.5 (3.3)	66.0 (8.3)	0.32
Married, n (%)	4 (36)	5 (46)	4 (100)	3 (75)	0.38
College education, n (%)	3 (27)	3 (27)	2 (50)	0 (0)	0.30
Self-reported race, n (%)					0.88
Asian	1 (9)	0 (0)	0 (0)	0 (0)	
Black	1 (9)	1 (9)	0 (0)	0 (0)	
White	9 (82)	9 (82)	4 (100)	4 (100)	
Other†	0 (0)	1 (9)	0 (0)	0 (0)	
Hispanic, Latino, or Spanish, n (%)	0 (0)	2 (18)	0 (0)	0 (0)	0.32
Ability to pay for basic living expenses, n (%)					0.82
Not hard at all	8 (73)	9 (82)	4 (100)	3 (75)	
Somewhat or very hard	2 (18)	2 (18)	0 (0)	1 (25)	
Prefer not to answer	1 (9)	0 (0)	0 (0)	0 (0)	
**Health-related Behaviors**					
Physical activity‡, n (%)					0.07
Inactive	2 (20)	3 (27)	0 (0)	1 (25)	
Insufficiently active	4 (40)	1 (9)	0 (0)	3 (75)	
Sufficiently active	4 (40)	7 (63)	4 (100)	0 (0)	
Current smoking, n (%)	0 (0)	0 (0)	0 (0)	0 (0)	-
Heavy alcohol use§, n (%)	4 (36)	0 (0)	1 (25)	2 (50)	0.11
**Self-reported Comorbidities, n (%)**					
Diabetes mellitus	3 (27)	2 (18)	2 (50)	0 (0)	0.38
Hypertension	3 (33)	5 (46)	3 (75)	3 (75)	0.38
Coronary artery disease	2 (18)	0 (0)	3 (75)	1 (25)	0.02
Congestive heart failure	1 (9.1)	0 (0)	0 (0)	0 (0)	0.64
Chronic obstructive pulmonary disease	2 (18)	0 (0)	0 (0)	0 (0)	0.30
Number of comorbidities, mean (SD)	2.0 (2.4)	1.0 (1.1)	2.8 (1.9)	1.8 (0.5)	0.34
**American Urological Symptom Index (AUASI) at Baseline, mean (SD)**					
Total score	21.5 (6.7)	19.1 (5.6)	17.7 (4.0)	21.2 (8.5)	0.72
Storage subscore	9.3 (2.8)	8.5 (2.2)	8.7 (2.9)	11.2 (3.5)	0.36
Voiding subscore	12.2 (5.9)	10.6 (4.3)	9.8 (2.1)	10.0 (5.5)	0.78
Urinary bother score	3.3 (1.3)	2.6 (1.7)	3.5 (1.7)	3.0 (0.8)	0.68
**Lower Urinary Tract Symptom Treatments at Baseline**					
Tamsulosin daily dose					0.13
0.4mg per day	9 (82)	9 (82)	3 (75)	1 (25)	
0.8mg per day	2 (18)	2 (18)	1 (25)	3 (75)	
Combination therapy (+ 5α-reductase inhibitor)	4 (36)	4 (36)	1 (25)	0 (0)	0.54

* Includes 4 individuals who attempted the n-of-1 protocol and dropped out during the 1-week placebo run-in due to worsening symptoms.

† Neither participant who selected this option provided additional information regarding their race.

‡ Calculated as minutes per week engaged in moderate to strenuous activity and was categorized as Inactive: 0 min/week; Insufficiently active: 1-149 min/week; Sufficiently active: 150+ min/week.

§ Alcohol use was tabulated as a composite value integrating alcohol consumption frequency ("How often do you have a drink?") and density ("How many standard drinks on a typical day, how often do you have ≥ 6 drinks on one occasion?") and a score ≥ 4 indicated a positive screen for heavy alcohol use.
‖ P-value calculated using ANOVA for continuous variables and χ^2 tests for categorical variables.

eTable 3. Change in quality of life, urinary bother, and tamsulosin nonadherence from baseline to follow-up after competition of the N-of-1 protocol among the 26 protocol completers.

Variable	Baseline	Follow-up	Change	P-value[†]
PROMIS-29* Physical Health summary score, mean (SD)	50.8 (8.8)	52.3 (7.7)	1.4 (5.7)	0.54
PROMIS-29* Mental Health summary score, mean (SD)	51.4 (7.1)	53.4 (6.8)	2.1 (4.8)	0.29
LURN SI-29 Global Urinary Bother, median (IQR)	3.0 (2.0)	2.0 (1.0)	-1.0 (2.0)	0.001
Perceived benefit from tamsulosin				0.72
None	1 (3.8%)	2 (8.0%)	-	
Little	8 (30.8%)	9 (36.0%)	-	
Much	17 (65.4%)	14 (56.0%)	-	
Satisfaction with chronic tamsulosin therapy				0.44
Much dissatisfied	1 (4.0%)	3 (12.0%)	-	
Little dissatisfied	3 (12.0%)	6 (24.0%)	-	
Little satisfied	6 (24.0%)	4 (16.0%)	-	
Much satisfied	15 (60.0%)	12 (48.0%)	-	

* Based on the PROMIS 29 v2.0 Profile.
[†] P-value calculated using *t*-tests for continuous variables and χ^2 tests for categorical variables.

eFigure 1. Categories of tamsulosin treatment response based on the upper bound of 95% CIs for individual-specific effect estimates.

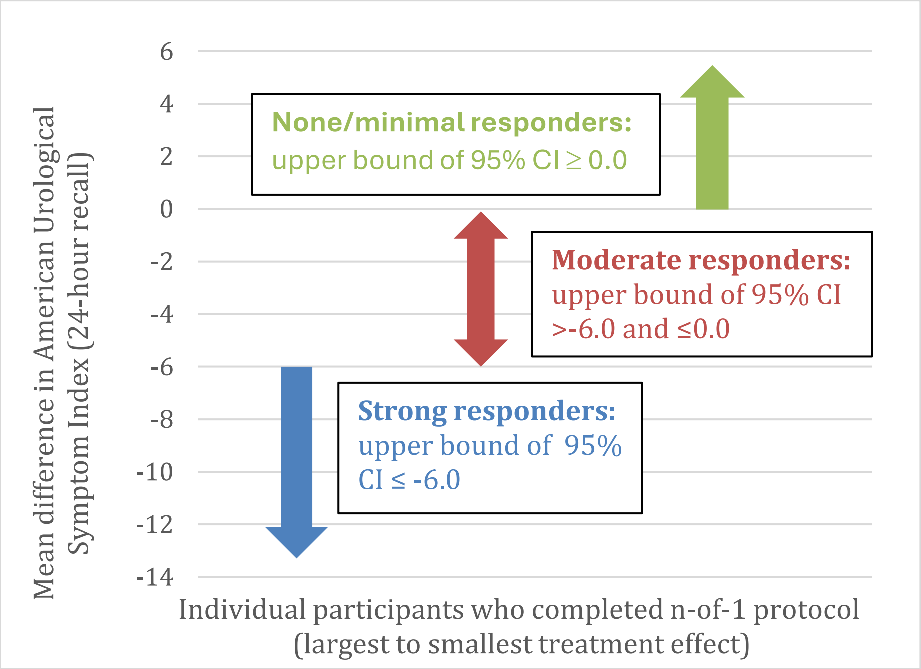

eFigure 2. Pharmacokinetics of tamsulosin elimination in older men receiving chronic tamsulosin therapy.

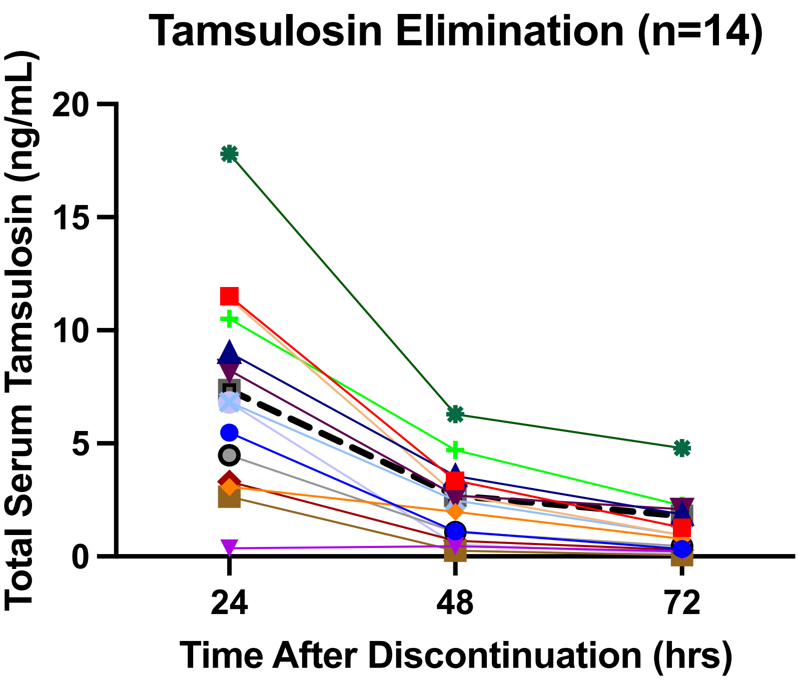

Participant Number	$T_{1/2}$ (hours)
P1	11.81
P2	15.10
P4	24.27
P5	14.56
P6	8.39
P7	21.03
P8	24.25
P9	13.68
P11	25.34
P12	13.34
P13	21.53
P14	16.93
P15	8.16
P16	16.80
Average	11.81

eFigure 3. Example of a report provided to a study participant with a strong tamsulosin response after completing the N-of-1 protocol.

Study Participant Report

Participant Name: John Doe

Dates of Participation: 1/22/2022 – 4/15/2022

PERSONAL

International Prostate Symptom Score (IPSS) Results

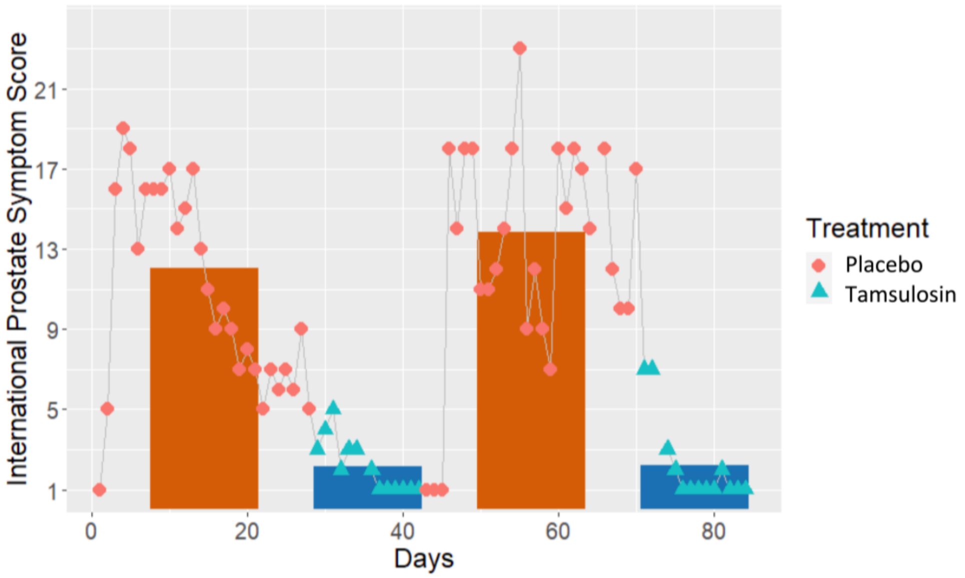

The **red** circles above represent the International Prostate Symptom Scores (IPSS) calculated from the surveys that you completed while taking placebo (sugar) pills during the study. The red bars represent the mean IPSS score during treatment periods when you were taking placebo pills. Overall, your mean IPSS score while taking placebo pills was 13.0 (standard deviation = 4.2).

The **blue** triangles represent the IPSS scores calculated from the surveys that you completed while taking tamsulosin pills during the study. The blue bars represent the mean IPSS score during treatment periods when you were taking tamsulosin pills. Overall, your mean IPSS score while taking tamsulosin pills was 2.2 (standard deviation = 1.8).

What should you do with this information?

Lower IPSS scores mean that you are reporting less severe urinary symptoms. Most men report satisfaction with medications for BPH if their IPSS score is 2 to 3 points lower compared to taking a placebo or no medication, although every man is different. Share this information with your doctor and discuss whether or not your tamsulosin medication is helping your urinary symptoms.

eReferences (Supplemental Methods Only)

1. Barry MJ, Fowler FJ, Jr., O'Leary MP, et al. The American Urological Association symptom index for benign prostatic hyperplasia. The Measurement Committee of the American Urological Association. *J Urol*. Nov 1992;148(5):1549-57; discussion 1564.
2. Lee AW, Kenfield SA, Wang EY, et al. Tracking Lower Urinary Tract Symptoms and Tamsulosin Side Effects Among Older Men Using a Mobile App (PERSONAL): Feasibility and Usability Study. *JMIR Form Res*. Dec 10 2021;5(12):e30762. doi:10.2196/30762
3. Wang EY, Breyer BN, Lee AW, et al. Perceptions of Older Men Using a Mobile Health App to Monitor Lower Urinary Tract Symptoms and Tamsulosin Side Effects: Mixed Methods Study. *JMIR Hum Factors*. Dec 24 2021;8(4):e30767. doi:10.2196/30767
4. McVary KT, Roehrborn CG, Avins AL, et al. Update on AUA guideline on the management of benign prostatic hyperplasia. *J Urol*. May 2011;185(5):1793-803. doi:10.1016/j.juro.2011.01.074
5. Barry MJ, Williford WO, Fowler FJ, Jr., Jones KM, Lepor H. Filling and voiding symptoms in the American Urological Association symptom index: the value of their distinction in a Veterans Affairs randomized trial of medical therapy in men with a clinical diagnosis of benign prostatic hyperplasia. *J Urol*. Nov 2000;164(5):1559-64.
6. O'Leary M P. Validity of the "bother score" in the evaluation and treatment of symptomatic benign prostatic hyperplasia. *Reviews in urology*. Winter 2005;7(1):1-10.
7. Cella D, Smith AR, Griffith JW, et al. A new outcome measure for LUTS: Symptoms of Lower Urinary Tract Dysfunction Research Network Symptom Index-29 (LURN SI-29) questionnaire. *Neurourology and urodynamics*. Aug 2019;38(6):1751-1759. doi:10.1002/nau.24067
8. Giuse NB, Koonce TY, Kusnoor SV, et al. Institute of Medicine Measures of Social and Behavioral Determinants of Health: A Feasibility Study. *Am J Prev Med*. Feb 2017;52(2):199-206. doi:10.1016/j.amepre.2016.07.033
9. Hays RD, Spritzer KL, Schalet BD, Cella D. PROMIS(®)-29 v2.0 profile physical and mental health summary scores. *Quality of life research : an international journal of quality of life aspects of treatment, care and rehabilitation*. Jul 2018;27(7):1885-1891. doi:10.1007/s11136-018-1842-3
10. Voils CI, Maciejewski ML, Hoyle RH, et al. Initial validation of a self-report measure of the extent of and reasons for medication nonadherence. *Medical care*. Dec 2012;50(12):1013-9. doi:10.1097/MLR.0b013e318269e121
11. Voils CI, King HA, Thorpe CT, et al. Content Validity and Reliability of a Self-Report Measure of Medication Nonadherence in Hepatitis C Treatment. *Dig Dis Sci*. Oct 2019;64(10):2784-2797. doi:10.1007/s10620-019-05621-7
12. Reeve E, Low LF, Shakib S, Hilmer SN. Development and Validation of the Revised Patients' Attitudes Towards Deprescribing (rPATD) Questionnaire: Versions for Older Adults and Caregivers. *Drugs & aging*. Dec 2016;33(12):913-928. doi:10.1007/s40266-016-0410-1
13. Astellas Pharma Inc. Flomax® (tamsulosin hydrochloride) [package insert]. U.S. Food and Drug Administration website. https://www.accessdata.fda.gov/drugsatfda_docs/label/2007/020579s020lbl.pdf. Revised July 2006. Accessed November 17, 2019.
