# Supplementary material for: Tamsulosin Deprescribing for Lower Urinary Tract Symptoms in Older Men: A Randomized Clinical Trial
Source: JAMA Netw Open. 2026 Jul 6;9(7):e2621639. doi: 10.1001/jamanetworkopen.2026.21639 (PMC13338806; doi:10.1001/jamanetworkopen.2026.21639)
Supplement: Supplement 2. — Trial Protocol [file jamanetwopen-e2621639-s002.pdf]

Placebo-controlled, Randomized, patient-Selected Outcomes N-of-1 triALs (PERSONAL): alpha-blockers for lower urinary tract symptoms

Protocol Version Number: 1.0
Protocol Version Date: 01/20/2023

Principal Investigator (Sponsor-Investigator)

Benjamin N. Breyer MD, MAS, FACS
University of California San Francisco
400 Parnassus Ave, Suite 610
San Francisco, CA 94131
Telephone: 415-221-4810
E-mail: Benjamin.Breyer@ucsf.edu

Co-Principal Investigators (Sponsor-Investigator)

Scott Bauer MD, ScM
University of California San Francisco
4150 Clement Street
Building 2, Room 135
San Francisco, CA 94121
Telephone: 415-221-4810
x24322
E-mail: Scott.Bauer@ucsf.edu

Stacey Kenfield, ScD
University of California San Francisco
550 16th Street
San Francisco, CA 94158
Telephone: 415-476-5392
E-mail: Stacey.Kenfield@ucsf.edu

Statisticians

Charles E. McCulloch, PhD
Kaiwei Lu, MS

Revision History

Version 1.0	01/20/2023

Proprietary and Confidential

The information in this document is considered privileged and confidential and may not be disclosed to others except to the extent necessary to obtain Institutional Review Board approval and informed consent, or as required by Federal and State laws. Persons to whom this information is disclosed should be informed that this information is privileged and confidential and that it should not be further disclosed

Abstract

Title	PlacEbo-controlled, Randomized, patient-Selected Outcomes N-of-1 triALs (PERSONAL): alpha-blockers for lower urinary tract symptoms
Study Description	This study will focus on determining if placebo-controlled N-of-1 deprescribing trials can identify older men who are unlikely to benefit from continuing chronic tamsulosin therapy for LUTS. We will also assess recruitment, retention, and completion rates for the study and other secondary outcomes among this population of older men receiving chronic tamsulosin therapy for LUTS to facilitate deprescribing decisions.
Study Intervention	Participants will start with a 1-week run-in period where they will use the PERSONAL Redcap surveys to track daily symptoms and side effects while taking only placebo study pills. Based on the pharmacokinetics and expected timeframe of symptomatic relief from tamsulosin (half-life=14 to 15 hours; steady state by the 5th day of daily dosing), all N-of-1 trials will have a duration of 12 weeks during which participants will complete the run-in and 2 cycles consisting of a pair of 2-week treatment periods (taking tamsulosin or placebo) separated by 1 week of wash-out with placebo. The order of treatment periods within a cycle will be random (e.g. ABAB, BABA, ABBA, or BAAB) according to pre-filled bubble packs given to participants during their orientation visit, but all patients will undergo 1 treatment of tamsulosin and 1 treatment of placebo during each of the 2 cycles. Participants will receive a placebo during wash-out periods between treatment periods and cycles, but they will be unaware of the order or duration of treatment periods or cycles to prevent self-correlating symptoms to specific treatments.<br><br>The PERSONAL Redcap will present participants with a daily questionnaire, accessible via smartphone, to track their symptoms. We chose to track the severity of lower urinary tract symptoms (LUTS) using an adapted version of the widely used American Urological Association Symptom Index (AUASI) with a 24-hour recall. This modified questionnaire includes daily questions regarding storage and voiding symptoms. All participants will also be presented a global urinary symptom bother question.
Study Population	Older men age 55-80 years based on the following *Inclusion criteria*:<br>Male sex at birth.<br>An ICD-10 diagnosis consistent with BPH<br>Has been taking Tamsulosin for at least 12 months with active prescription from a urologist<br>No history of urinary incontinence, acute urinary retention, recurrent urinary tract infections, obstructive kidney disease, or urethral stent<br>Able to speak and complete questionnaires in English.<br>Have an iOS or Android smartphone
Primary Objective	To determine if placebo-controlled N-of-1 deprescribing trials can identify older men who are unlikely to benefit from continuing chronic tamsulosin therapy for LUTS by assessing differences in daily urinary symptom severity between treatment with tamsulosin and placebo.

Secondary Objectives	• To describe the recruitment timeframe, study retention, and questionnaire completion rates<br>• To describe patient characteristics at baseline related to the condition and their medication use<br>• To describe adverse events during the study<br>• To describe the magnitude of difference in quality of life and urinary bother between treatment with tamsulosin and placebo
Recruitment Methods	We will use a mix of secure electronic health record messaging and phone calls to invite patients to enroll.
Sample Size	This feasibility study will enroll at least 20 older men with LUTS/BPH.
Duration of Study Participation	All study participants will be followed for about 4 months from enrollment (screening/recruitment period, 3-month intervention).

List of Abbreviations

5-ARI	5 Alpha-Reductase Inhibitor
AE	Adverse Event
AUASI	American Urological Association Symptom Index
BPH	Benign Prostatic Hyperplasia
CI	Confidence Interval
CRC	Clinical Research Coordinator
CRF	Case Report From
CTCAE	Common Terminology Criteria for Adverse Event
EHR	Electronic Health Record
HIPAA	Health Information Portability and Accountability Act
ICD-10	International Classification of Disease, Tenth Revision
ICF	Informed Consent Form
IPSS	International Prostate Symptom Score
IRB	Institutional Review Board
LURN	Lower Urinary Tract Dysfunction Research Network
LUTS	Lower Urinary Tract Symptoms
MPI	Multiple Principal Investigator
PCORI	Patient Centered Outcomes Research Institute
PHI	Protected Health Information
PI	Principal Investigator
PSSUQ	Post-Study System Usability Questionnaire
RCT	Randomized Controlled Trial
rPATD	Revised Patients' Attitudes Towards Deprescribing
SAE	Serious Adverse Event
SD	Standard Deviation
SOM	School of Medicine
UCSF	University of California, San Francisco
UTI	Urinary Tract Infection

Table of Contents

ABSTRACT… 2
LIST OF ABBREVIATIONS...4
TABLE OF CONTENTS 5
1 INTRODUCTION 7
1.1 *Background: LUTS/BPH 7*
1.2 *Background: Alpha-blocker deprescribing 7*
1.3 *Risk/Benefit Assessment 8*
2 STUDY OBJECTIVES 9
3 STUDY DESIGN 10
4 SELECTION AND ENROLLMENT OF PARTICIPANTS 11
4.1 *Eligibility Criteria 11*
4.2 *Recruitment and Screening... 11*
4.3 *Participant Registration 12*
5 SCREENING, CONSENT, AND RANDOMIZATION 13
5.1 *Screening ……………………………………………………………………………13*
5.2 *Informed Consent 13*
5.3 *Randomization and Blinding 13*
6 STUDY PROCEDURES AND ASSESSMENTS 14
6.1 *Schedule of Activities.. 14*
6.2 *Assessments………………………………………………………………………... 15*
6.3 *Questionnaire Data……………………………………………………15*
6.4 *Study Assessments by Visit…………………………………………..16*
7 INTERVENTION 18
8 ENDPOINTS……………………………………………………………………………19
9 STATISTICAL CONSIDERATIONS ………………………………………………………20
9.1 *Power and Sample Size.. …20*
9.2 *Statistical Analysis Plans .... …..20*
10 ADVERSE EVENTS AND REPORTING REQUIREMENTS..22
10.1 DEFINITIONS………………………………………………………………22
10.2 EXPECTEDNESS…………………………………………………………22
10.3 ATTRIBUTION………………………………………………………………22
10.4 SEVERITY……………………………………………………………………23
10.5 REPORTING REQUIREMENTS……………………………….…………….23
11 PROTOCOL VIOLATION AND WITHDRAWAL OF PARTICIPANTS………………………………25
11.1 PROTOCOL VIOLATION………………………..……………………….25
11.2 WITHDRAWAL OF PARTICIPANTS…………………………………………25
12 DATA MANAGEMENT AND MONITORING…………………………..…………………26
12.1 INDIVIDUALS OVERSEEING DATA MANAGEMENT………..………………26
12.2 CASE REPORT FORMS AND SOURCE DOCUMENTS………...…………....26
12.3 DATA MANAGEMENT PROCEDURES………..……………………………26
12.4 DATA QUALITY CONTROL AND REPORTING. ……….……………………..26
12.5 DATA AND SAFETY MONITORING PLAN………..……………………………27

13 PROTECTION OF HUMAN SUBJECTS……………………………………………….…………..28
13.1 INFORMED CONSENT………..……………………….………………….28
13.2 POTENTIAL RISKS………..………………………..………………..28
13.3 POTENTIAL BENEFITS………..……………………………………..…. 28
13.4 ALTERNATIVES………..…………………………………………..28
13.5 CONFIDENTIALITY………..…………………………………………..29
13.6 VOLUNTARINESS OF RESEARCH PARTICIPATION……………………………..29
13.7 PATIENT PRIVACY………..…………………………...…………29
14 STUDY MANAGEMENT………..……………………………………..………30
14.1 *Pre-study Documentation* .... .30
14.2 *Institutional Review Board Approval* 30
14.3 *Informed Consent* 30
14.4 *Changes in the Protocol* 30
14.5 *Record Retention* 30
14.6 *Publications* ……………………………………………………………………31
15 REFERENCES 32

1.0 INTRODUCTION

1.1 Background: Lower urinary tract symptoms (LUTS) comprise a syndrome of overlapping symptoms that occur when urine is being generated and stored it in the bladder (i.e., *storage* LUTS, such as urgency, daytime frequency, nocturia, etc.), or during the initiation and process of urination (i.e., *voiding* LUTS, such as weak stream, straining, incomplete voiding, etc.).[1] More than 30% of men will develop clinically significant LUTS in their lifetime, and the majority of male LUTS, including both storage and voiding subtypes, are attributed to benign prostatic hyperplasia (LUTS/BPH).[2] In addition to worse health-related quality of life,[3-7] older men with LUTS/BPH have an increased risk of new mobility impairment, falls, fractures, disability, and death.[8-11] In 2000, the estimated direct costs of treating LUTS/BPH in the United States were $1 billion and increased to $4 billion after including indirect costs.[2,12] Since then, the cost of caring for men with LUTS/BPH has increased dramatically.[13,14]

Because more than 80% of men develop histologic evidence of BPH on autopsy by age 80 years,[15] prostate-centric therapies targeting bladder outlet obstruction have dominated the pharmaceutical and surgical treatment landscape for LUTS/BPH. However, growing evidence suggests that older man are more likely to suffer from LUTS caused by systemic, non-prostatic conditions that are not targeted by existing interventions, such as obstructive sleep apnea, kidney disease, or heart failure.[16,17] This is compounded by the failure of current diagnostic tests to accurately identify the specific cause of LUTS/BPH.[18,19]

One consequence of our limited diagnostic accuracy for LUTS/BPH is that older men are empirically prescribed medications that target prostatic smooth muscle and prostate enlargement at significantly higher rates than younger men with the same diagnosis (the new prescription rate for men with LUTS/BPH ages 50-59 years increases from 15 to 32 per 100 person-years for men ages 60-64 years), and this trend is increasing over time.[20] However, these medications have modest efficacy on LUTS severity[21] and have potentially harmful side effects in older men (e.g., orthostatic hypotension and dizziness, falls, fractures, depression),[22-28] leading to low adherence and high rates of discontinuation.[29,30] In addition to being particularly susceptible to the harmful side effects of these medications, older men with LUTS/BPH have lower levels of physical activity, increased obesity and metabolic syndrome, and increased frailty,[4,7,31-33] which further increases their risk of developing poor clinical outcomes.

1.2 Background: Alpha blocker deprescribing

Despite widespread use, the impact of α1-blockers compared to placebo on LUTS is small.[34] Clinical studies suggest that many patients, who are on α1-blocker monotherapy or combination therapy with 5-alpha-reductace inhibitors (5-ARIs), can discontinue α1-blocker therapy after initial improvement without the need to restart treatment. [35-37] Harms of α1-blockers, such as orthostatic hypotension and dizziness which lead to falls and fractures, have led to recommendations that they be used with caution in older men.[38,39] A recent study by Renoncourt *et al* suggested that over 79% of patients taking α1-blockers may be doing so inappropriately.[39] Another study by Edelman *et al* showed that most men would be willing to stop taking α1-blockers at the request of their doctor. [40] In the setting of modest benefits and known harms, a more personalized and patient-centric approach is needed to ensure that only men in whom benefits outweigh the harms continue to receive chronic α1-blocker therapy.

1.3 Risk/Benefit Assessment

Our eligibility criteria and screening procedures are established to exclude individuals for whom the study is not appropriate. Per the exclusion criteria, this includes patients with specific urologic or psychiatric conditions. After obtaining participant consent, the screening process will include verification of these factors by the clinician. This multi-gated comprehensive approach should systematically identify and screen out any individual for whom this study is not indicated. There are no direct benefits to the participants (financial compensation is purposely not presented as a benefit), except as to their feelings of being involved as participants in an important research study. Additionally, patients will learn about their results regarding response to tamsulosin which may lead to better informed decisions of whether to continue the medication.

This study will provide valuable insights into the feasibility of tamsulosin deprescription across a diverse patient population. The potential public health benefit to society in this study could be large, as we are targeting a common condition in older men (one in three older men develop LUTS/BPH in their lifetime). Our overarching goal is to build the evidence needed for a full-scale efficacy trial testing deprescription, thus this research has great potential to change the paradigms for LUTS/BPH management. Scientific and clinical knowledge gained from this study could be extremely useful to practicing clinician, individuals, policy makers, insurers, and public health planners developing interventions to prevent or treat LUTS/BPH. Thus, given the importance of knowledge to be gained and the anticipated benefit to research participants and others, the risks to subjects are reasonable.

2.0 STUDY OBJECTIVES

Primary Objective Aim 1

To determine if placebo-controlled N-of-1 deprescribing trials can identify older men who are unlikely to benefit from continuing chronic tamsulosin therapy for LUTS

Secondary Objective(s):

Aim 2: To describe the recruitment timeframe, study retention, and questionnaire completion rates.

Aim 3: To describe patient characteristics at baseline related to the condition and their medication.

Aim 4: To describe adverse events during the study.

Aim 5: To describe the magnitude of difference in quality of life and urinary bother between treatment with tamsulosin and placebo

3.0 STUDY DESIGN

Overview

We propose to conduct a 12-week double-blind, placebo-controlled, multiple crossover (N-of-1) randomized clinical trial study of at least 20 older men taking chronic tamsulosin therapy for urinary symptoms due to benign prostatic hyperplasia (BPH). During the 12-week study, all men will receive multiple 2-week treatment blocks on tamsulosin or placebo. Men ages age 55-80 years who speak, read and write English with an ICD-10 diagnosis of BPH, at least 12 months of chronic tamsulosin therapy for BPH-related urinary symptoms, who have a personal smartphone, and are willing to self-report urinary symptoms and adverse events will be recruited from several urology clinics at the University of California, San Francisco.

4.0 SELECTION AND ENROLLMENT OF PARTICIPANTS

To ensure we recruit a diverse sample, eligible participants will be identified by the electronic health record at UCSF Health.

4.1 Eligibility Criteria

Inclusion Criteria

In order to be eligible to participate in this study, an individual must meet all of the following criteria:

1. Urology patient at UCSF with ICD-10 diagnosis of BPH
2. Must own Android or iPhone smartphone
3. Taking tamsulosin for urinary-related symptoms for at least 12 months
4. Able to speak and read English
5. Male 55-80 years old of age at telephone screening.
6. Written informed consent (and assent when applicable) obtained from subject or subject' s legal representative and ability for subject to comply with the requirements of the study
7. Willing to receive electronic PERSONAL daily intake surveys for 3 months
8. Willing to self-report urinary symptom and adverse event data at specified frequency
9. Have home WiFi access.
10. Patients with history of prostate cancer may be enrolled but is not required
11. Patients with history of kidney stones may be enrolled but is not required

Exclusion Criteria

An individual who meets any of the following criteria will be excluded from participation in this study:

1. International Prostate Symptom Score <5 or >25 .
2. Current participation in any other mobile app-based clinical study.
3. Planning to relocate from area within the study duration.
4. Impaired vision that could limit the use of the mobile apps (participant-reported).

4.2 Recruitment and Screening Methods

The recruitment and screening procedures outlined below present no more than minimal risk to

the privacy of the participants who are screened, and a screening log containing minimal patient health information (PHI) will be maintained.

Potentially eligible patients will be identified through the electronic health record (EHR) by UCSF Research Participant Services. University of California, San Francisco (UCSF) patients who meet inclusion criteria based on data available in the EHR and who have previously agreed to be contacted by UCSF Research Participant Services will receive a secure EHR message informing them about the study and inviting them to contact research staff if interested in participating, as we have done before.

If needed, based on recruitment rates, we will post flyers and contact clinicians at UCSF urology clinics who are willing to sponsor clinic-specific recruitment efforts. If needed, we will also engage with community partners to disseminate study recruitment information and flyers.

If an individual is interested in learning more about the study, research staff will meet with the potential participant by phone to discuss the study and screening procedures. If the patient is interested in participating in the study, he will be asked to respond to the screening questions by phone to further assess eligibility.

4.3 Participant Registration

A written or electronic informed consent form (ICF) must be signed or acknowledged before any study-specific assessments are initiated. A copy of the signed ICF will be given to the participant and a copy will be filed in the medical record. The original will be kept on file with the study records.

All participants consented to the study will be registered in OnCore®, the UCSF Helen Diller Family Comprehensive Cancer Center Clinical Trial Management System. The system is password protected and meets HIPAA requirements.

5.0 SCREENING, CONSENT, RANDOMIZATION

5.1 Screening

Eligible patients at the UCSF Mission Bay and Parnassus sites will be identified through a medical records query using MyChart Recruitment.

My Chart Recruitment:
MyChart (Apex) conducts a search for patients based on the study's inclusion and exclusion criteria. This is a completely computer-aided search, meaning the computer—and not a person--searches patient charts. When a patient is identified as potentially eligible, they receive an email from MyChart that says to log in to MyChart to read about a study they might be interested in. The email is short and is the same for every recipient—there is no patient-specific, study-specific or disease information in it.

When the patient logs into MyChart, there is a new "Research" tab with template information about participating in research and how to opt out of receiving recruitment messages. Then, the patient can click through to learn about a specific study they may be eligible for. The patient has the option of clicking a link/button to let the study team know that they are interested in learning more about the study. Only if the patient takes this action will the study team receive information about the patient. If the patient clicks "No thanks" or simply does not respond, they will not be contacted by the study team, they won't receive any follow-up emails from MyChart about this study, and their information will not be shared with the study team.

If the patient indicates interest, our research staff will reach out to eligible patients for a telephone screening to determine eligibility. Phone screening will determine if the participant owns a personal smartphone, participant age, abbreviated medical history, and specifically checking for diagnosis of benign prostatic hyperplasia (BPH) and use of tamsulosin for the past 12 months for urinary symptoms. Study visits and procedures will be described, and eligible persons will be invited to attend a baseline visit held remotely. Likely eligible individuals will be asked to provide informed consent using an IRB-approved electronic informed consent process before any study activities take place. At the conclusion of the telephone screening, participants will be sent a secure email through the REDCap database with links to the study mobile application, with instructions to download the application.

5.2 Informed Consent

All study participants must willingly consent after being informed of the study activities and procedures to be followed, the experimental nature of the intervention, alternatives, potential benefits, side effects, risks, and discomforts. Human protection committee approval of this protocol and its consent form are required, as well as any material that is seen by study participants. Informed consent is required before any study-specific procedures are performed. We will used UCSF DocuSign to obtain an electronic signature of consent, and paper version as alternative method if needed.

5.3 Randomization and blinding

This will be a double-blinded, placebo-controlled, N-of-1 RCT. The randomization scheme will be computer-generated by the study statistician without participant contact. Participants will be randomized into 1 of 4 treatment schedules with 2 cycles each containing treatment blocks of tamsulosin and placebo (e.g. ABAB, BABA, ABBA, or BAAB). Data analysts, and investigators will

remain blinded to participants' randomization arm. Primary outcomes will be collected using self-administered questionnaires without risk of unblinding. Intervention status and any variables related to intervention status will be recorded and stored in a separate database by unblinded Clinical Research Coordinator (CRC).

6.0 STUDY PROCEDURES and ASSESSMENTS

6.1 Schedule of Activities (Table 1)

Assessments/Procedures	Screening	Study Period												Post-treatment
Timeline	Screening	Run-in	Period 1a		Wash out	Period 1b		Wash out	Period 2a		Wash out	Period 2b		
Week (W)		W1	W2	W3	W4	W5	W6	W7	W8	W9	W10	W11	W12	W14
Informed consent	X													
Randomization	X													
Clinical History (medical, medication use, symptoms)	X													
Socio-demographics	X													
Questionnaires: medication adherence, urinary satisfaction, health-related quality of life	X													X
Placebo only (daily)		X			X			X			X			
Randomized to Placebo or Tamsulosin			X	X		X	X		X	X		X	X	
Daily Questionnaires (IPSS/AUASI, adverse events)		X	X	X	X	X	X	X	X	X	X	X	X	
Continuous monitoring for adherence		X	X	X	X	X	X	X	X	X	X	X	X	
Review Results with patient														X
Other adverse event reporting		X	X	X	X	X	X	X	X	X	X	X	X	X

Abbreviations: IPSS International Prostate Symptom Score; AUASI American Urological Association Symptom Index

6.2 Assessments

6.2.1 Medical screening

The medical screening process is described in Section 5.1.

6.2.2 Demographic/Clinical Information

Demographic information (e.g., date of birth, race, ethnicity, marital status, education) will be recorded at Screening. Clinically relevant medical history, including history of current disease, other pertinent clinical conditions, and information regarding underlying diseases will be recorded at Screening.

The following items should be reported:

- **Medical History:** includes medical conditions that are commonly associated with LUTS, other urologic history, psychiatric history, health-related behaviors, and cardiovascular comorbidities.
- **Comprehensive BPH/LUTS treatment history:** current/past/never use of BPH/LUTS medications, procedures, behavioral interventions (e.g., pelvic floor physiotherapy, bladder training, timed voiding, double voiding, diet)

6.3 Questionnaire Data

Once eligibility is determined, participants will complete a set of questionnaires, that is estimated to take approximately 60 minutes. During week 14 (post-study), a subset of questionnaires will be repeated. During the study period, a smaller questionnaire assessing lower urinary tract symptoms will be administered daily via the phone app.

Questionnaires to be completed for primary and exploratory outcome assessment include:

- **International Prostate Symptom Score (IPSS)/American Urological Association Symptom Index (AUASI)[41]:** We will assess LUTS severity via the 7-item IPSS/AUASI, which is the most widely used male LUTS instrument in both clinical and research settings. IPSS total score is continuous (range: 0 to 35) and has clinically relevant categories (0-7, 8-19, 20-35) indicating no/mild, moderate, and severe LUTS, respectively, as well as validated storage and voiding subscores.[42] The minimally important difference is 2-3 points for the IPSS, based on therapeutic response to BPH surgery.[43] We will administer the original IPSS/AUASI at baseline and follow-up as well as daily using a modified daily version with a recall period of 24 hours (versus 1 month) to allow for more frequent assessments.

- **Revised Patients' Attitudes Towards Deprescribing (rPATD)[44]**: We will assess patient attitudes toward deprescribing using this standard set of 22 questions each graded on a 5-point Likert scale. The subdomains include burden (perceived burden of routine medication taking), appropriateness (perceived benefits/harms of the medication), concerns about stopping (concerns related to stopping the medication), and involvement (perceived involvement in care and understanding of the medication).

- **Perceived benefit from tamsulosin:** We will assess patient perceived benefit from tamsulosin by asking a single question of perceived benefit in which patients can choose between 3 options: “none”, “little”, and “much”.

- **Satisfaction with chronic tamsulosin therapy:** This will be assessed with a question with 4 individual option choices: “much satisfied”, “little satisfied”, “little dissatisfied”, and “much dissatisfied”.

- **Voils DOSE-Nonadherence measure[45]:** We will assess patient's baseline tamsulosin medication adherence using this questionnaire which consists of two sections. The first summarizes the frequency of missed doses and the second section assesses the cause of the missed dose.

- **PROMIS-29 Profile 2.0:** The Patient-Reported Outcomes Measurement Information System (PROMIS®) is a National Institutes of Health initiative to develop state-of-the-science self-report measures to assess functioning and well-being in physical, mental and social domains of health. PROMIS measures are potentially useful to screen for disability, identify health care disparities, enhance communication between patients and clinicians, and improve population health.

6.4 Study Assessments by Visit

Screening Prior to Baseline:

1) Eligible patients at the UCSF Mission Bay and Parnassus sites will be identified through a medical records query and sent an email through MyChart to participate in the study. Patients who are interested will e-confirm their interest.
2) Research staff will reach out to interested patients and conduct a telephone screening call to determine eligibility.
3) Study visits and procedures will be described and eligible persons will be invited to attend the baseline visit.

Baseline (Orientation) Visit

Consented participants who completed the appropriate assessments prior to baseline will complete the following procedures at the baseline visit:

1) If an in-person baseline visit is not feasible, it will be held remotely over a Zoom Conference.
2) Study coordinators will obtain written informed consent from eligible participants.
3) Participants will be asked to complete a self-administered questionnaire. A complete medical history will be ascertained, including smoking and alcohol use.
4) We will also be measuring patient attitudes toward deprescribing using questionnaires including the Revised Patients' Attitudes Towards Deprescribing (rPATD), Voils Dose-nonadherence measure, and satisfaction with tamsulosin.
5) At the end of the orientation visit, research staff will provide verbal and written instructions for the study. Research staff contact information will be provided for reporting severe or concerning symptoms for the duration of the study.

End of study remote visit

1) After finishing the 12 weeks of the study, participants will be contacted to assess adherence to the program and complete a follow-up questionnaire.
2) Participants will be asked to complete self-administered questionnaires via REDCap. These will include the same questionnaires measuring patient attitudes toward deprescribing using tools including the Revised Patients' Attitudes Towards Deprescribing (rPATD), Voils Dose-nonadherence measure, and satisfaction with tamsulosin.
3) A study clinician will review the patients' individual results with them in detail.

7.0 Intervention Period

7.1 Intervention

All N-of-1 trials will have a duration of 12 weeks during which participants will complete the run-in and 2 cycles consisting of a pair of 2-week treatment periods (taking tamsulosin or placebo) separated by 1 week of wash-out on placebo. The order of treatment periods within a cycle will be random (e.g. ABAB, BABA, ABBA, or BAAB).

Participants will receive a bubble pack with 11 weeks of tamsulosin (at their previously prescribed dose of 0.4mg or 0.8mg) or matching placebo and will be instructed to start taking the study medications after successfully completing the 1-week run-in period.

Participants will start with a 1-week placebo run-in period where they will use the daily symptom questionnaires, accessible via smartphone or computer to track daily symptoms and adverse events. Participants will also receive a placebo during wash-out periods between treatment periods and cycles, but they will be unaware of the order or duration of treatment periods or cycles to prevent self-correlating symptoms to specific treatments.

The PERSONAL REDCap project will present participants with a daily questionnaire, accessible via smartphone, to track their symptoms. We chose to track the severity of LUTS using a modified version of the widely used IPSS/AUASI. This modified questionnaire includes daily questions regarding storage and voiding symptoms. All participants will also be presented a daily global urinary symptom bother question. Medication adherence, global urinary satisfaction questions, and health-related quality of life will be assessed as baseline and at the end of the study.

Participants will view a graphical representation of their responses during tamsulosin and placebo treatment at the end of the study. To maximize adherence to daily questionnaires, participants will be contacted via email or phone if they do not complete the daily symptom questionnaire for more than 3 consecutive days during their N-of-1 trial. Then, a PERSONAL clinician will review N-of-1 trial results with the participant at the end of the study.

8.0 ENDPOINTS (BY AIM)

Aim 1: To determine if placebo-controlled N-of-1 deprescribing trials can identify older men who are unlikely to benefit from continuing chronic tamsulosin therapy for LUTS

- change in urinary symptoms measured with the 24-hour recall adapted IPSS score between treatment with tamsulosin and placebo

Aim 2: To describe the recruitment timeframe, study retention, and questionnaire completion rates.

- recruitment timeframe (months)
- study completion rate (goal >70% of participants)
- questionnaire completion rate (% completing >50% of daily questionnaires)

Aim 3: To describe patient characteristics at baseline related to the condition and their medication

- urinary bother

- Tamsulosin satisfaction
- Tamsulosin adherence
- patient attitudes towards deprescribing

Aim 4: To assess adverse events during the study

- % reporting any adverse events

Aim 5: To describe the change in quality of life and urinary bother after a N-of-1 deprescribing trial

- change in quality of life measured with PROMIS-29 and change in the LURN SI-29 urinary bother question before and after the N-of-1 deprescribing trial

9.0 STATISTICAL CONSIDERATIONS

9.1 Power and Sample Size

Sample size justification. We determined sample size based on expected attrition rates. Based on our previously published protocol paper and prior mobile health studies [46,47]we expect to fail to meet our study goals (i.e., recruit and retain sufficient participants, achieve sufficient daily questionnaire and N-of-1 trial completion rates, and achieve sufficient "usefulness" scores among participants) at least 10% of the time. Therefore, with a sample size of 20 participants, we will have 90% power to observe at least one failure during this feasibility study.[48,49]

9.2 Statistical Analysis Plans

We will use multivariable adjusted linear mixed models with individual-specific intercepts and treatment effects and an unstructured variance-covariance matrix to estimate variation in daily AUASI score and daily summary side effect score. Treatment, day, and period will be included as independent variables. Individual-specific intercepts and treatment effects will allow for the estimation of individual-specific response to treatment for both AUASI score (shown below):

$$AUASI_{ij} = \beta_1 + \beta_2 Treatment_{ij} + \beta_3 Day_{ij} + \beta_4 Period_{ij} + b_{i1} + b_{i2} Treatment_{ij} + \varepsilon_{ij}$$

We will use individual-specific effect estimates of tamsulosin treatment on daily AUASI to define strong responders (upper bound of 95% confidence interval ≤ -6.0), moderate responders (upper bound of 95% confidence interval >-6.0 and ≤0.0), and minimal/non-responders (upper bound of 95% confidence interval ≥0). We selected the minimum upper bound of 95% confidence interval for strong responders based on the mean expected effect size of BPH surgery. We selected the minimum upper bound of 95% confidence interval for moderate responders to identify individuals with a statistically significant decrease in AUASI. Minimal/non-responders will be defined as those without 95% confidence intervals that include 0, suggesting no statistically significant difference between treatments, or those with a lower bound of 95% confidence interval ≥0. To visualize individual treatment effects, we will create a bar graph with the mean effect of tamsulosin on daily AUASI and 95% confidence interval for each participant, overall and by important subgroups such as baseline LUTS severity or age. We will then repeat this process for medication side effects.

To determine if there are carryover effects, we will include a variable indicating the sequence of each period (tamsulosin then placebo or placebo then tamsulosin) in the linear mixed model. To

evaluate whether treatment effect is correlated with baseline characteristics, we will calculate the correlation between the random intercept and random treatment effect and used a likelihood ratio test to compare models with an unstructured covariance matrix versus an independent covariance matrix.

We will use descriptive statistics to summarize the feasibility outcomes. In general, frequency distribution and percentage will be used to summarize categorical variables and median with interquartile range (IQR) to describe continuous variables. 95% confidence intervals (CIs) will be used to quantify the precision of these performance estimates.

We will pre-designate drop-out rates of <15%, 15-25%, and >25% as indicating optimal, acceptable, and inadequate participant retention during the 12-week intervention period. Among non-dropouts, we will consider completion of >85%, 75-85%, and <75% of questionnaire completion to indicate optimal, acceptable, and inadequate intervention adherence, respectively.

10.0 ADVERSE EVENTS AND REPORTING REQUIREMENTS

10.1 Definitions

An adverse event (AE) is any untoward or unfavorable medical occurrence in a study participant, including any abnormal sign (e.g., abnormal physical exam or laboratory finding), symptom, or disease, temporally associated with the participants' involvement in the research, whether or not considered related to participation in the research. Medical conditions or diseases present before starting study interventions should only be considered adverse events if they worsen after starting the interventions.

A serious adverse event (SAE) is any AE that results in death, is life threatening, or places the participant at immediate risk of death from the event as it occurred, requires or prolongs hospitalization, causes persistent or significant disability or incapacity, results in congenital anomalies or birth defects, or any other important event judged by the investigators to jeopardize the safety of a participant based upon appropriate medical judgment.

An unexpected problem is defined as any incident, experience, or outcome that meets all of the following criteria: 1) is unexpected, in terms of nature, severity, or frequency, given the research procedures described in the protocol and the characteristics of the study population; 2) is related or possibly related to participation in the research; and 3) suggests that the research places participants or others at a greater risk of harm (including physical, psychological, economic, or social harm) than was previously known or recognized.

10.2 Expectedness

An adverse event or suspected adverse reaction is considered "unexpected" if it is not listed above or included in the following list:

- Dizziness
- Headache
- Erectile dysfunction
- Decreased libido
- Rhinitis
- Fatigue
- Insomnia
- Diarrhea
- Constipation
- Nausea

- Back pain
- Worsening lower urinary tract symptoms

10.3 Attribution

A suspected adverse reaction means any adverse event for which there is reasonable possibility that the intervention caused the adverse event. For the purposes of safety reporting, “reasonable possibility” means there is evidence to suggest a causal relationship between the intervention and the adverse event.

The investigative team will assign attribution of the possible association of the event with the study intervention using the following definitions:

Unrelated (nonattributable) to the deprescription intervention: The adverse event is *clearly not related* or is *doubtfully related* to the deprescription intervention

Related (possibly attributable or attributable) to the deprescription intervention: The adverse event *may be related,* is *likely related,* or is *clearly related* to the deprescription intervention

10.4 Severity

Signs or symptoms shall be graded and recorded by the Investigator according to the Common Terminology Criteria for Adverse Events (CTCAE) (use latest version at initiation of clinical trial). When specific adverse events are not listed in the CTCAE, they are to be graded as mild, moderate, severe, or life-threatening according to the following grades and definitions:

AE Severity Grading

**Severity (Toxicity Grade)**	**Description**
Grade 1	Mild; asymptomatic or mild symptoms; clinical or diagnostic observations only; intervention not indicated
Grade 2	Moderate; minimal, local or noninvasive intervention indicated; limiting age- appropriate instrumental activities of daily living (ADL)
Grade 3	Severe or medically significant but not immediately life-threatening; hospitalization or prolongation of hospitalization indicated; disabling; limiting self-care ADL
Grade 4	Life-threatening consequences: urgent intervention indicated
Grade 5	Death related to AE

10.5 Reporting Requirements

For this study, new AEs or SAEs will be considered reportable any time after the Baseline Visit until 7 days (for non-serious AEs) or 28 days (for SAEs) after the last day of study participation. All events should be followed to their resolution, until the Investigator assesses them as stable, irreversible, or until the study participant is lost to follow-up, whichever comes first.

The study coordinator will record all reported events in the adverse event log (including subject's name, date, and event description). All SAEs occurring during the study must be reported to the appropriate study investigator within 24 hours of their knowledge of the event. The study PI will consult with the co-investigators on the action to be taken. This action and date of implementation will also be recorded in the adverse event log. The entire investigative team will participate in classifying events as AEs, SAEs, or unexpected problems, as well as 'non-attributable', 'possibly attributable', or 'attributable' to the proposed study.

The study will follow UCSF Reporting Requirements: UCSF requires submission of Adverse Events that qualify as: Any unexpected, physical, psychological or social research-related event which is definitely, probably or possibly related to the study; where the risk is not included, or exceeds the nature, severity, or frequency described in the protocol, study consent form, or other study information previously reviewed and approved by the IRB.

An unexpected AE also includes any AE that meets any of the following criteria:

- Results in subject withdrawal from study participation
- Due to an overdose of study medication
- Due to a deviation from the IRB approved study protocol

11.0 PROTOCOL VIOLATION AND WITHDRAWAL OF PARTICIPANTS

11.1 Protocol Violation

A protocol violation occurs when a study participant or Investigator fails to adhere to specific protocol requirements affecting the inclusion, exclusion, study participant safety and primary endpoint criteria. Protocol violations for this study include, but are not limited to:

- Randomization of a participant who does not meet the inclusion/exclusion criteria
- Inappropriate delivery of experimental or control interventions to participants in the wrong study arm
- Use of prohibited co-interventions during the study treatment period
- Any other deviation that presents significant risk or safety concerns to the study participant
- The investigative team will determine if a protocol violation should result in withdrawal of a study participant.

11.2 Withdrawal of participants

Participants are free to withdraw from participation in the study at any time upon request. If a participant withdraws from the study, any data collected on him up to that point in the study will go forward for study analysis. This information will be stated in the participant information leaflet. Reasons for stopping the intervention will be recorded and reported. When appropriate, outcome and follow-up data will be obtained, unless the participant specifically declines further follow-up.

An investigator may discontinue a participant from the study for the following reasons:

- Significant study intervention non-compliance (compliance is an aspect of the study objectives)
- Lost-to-follow up; unable to contact participant
- Any event, medical condition, procedure, surgery, or situation occurs such that continued collection of follow-up study data would not be in the best interest of the participant or might require an additional treatment that would confound the interpretation of the study

12.0 DATA MANAGEMENT AND MONITORING

12.1 Individuals Overseeing Data Management

A Clinical Research Coordinator (CRC) will be assigned to the study. The responsibilities of the CRC include screening and enrolling subjects; data collection, abstraction, entry, and reporting; communicating with participants; scheduling study participant visits; regulatory monitoring; and problem resolution and prioritization. The data collected for this study will be entered into a secure database (REDCap). Source documentation will be available to support the computerized participant record. The principal investigator will maintain ultimate responsibility for the clinical trial.

12.2 Case Report Forms and Source Documents

Participant data will be collected using protocol-specific case report forms (CRF). Source documentation will include only those documents containing original forms of data, including clinic charts, shadow files, hospital charts, and clinician notes. Data recorded directly on the CRFs designated as source documents (i.e., no prior written or electronic record of data) will be considered source data. All other data recorded on the CRFs will not be considered source documentation.

12.3 Data Management Procedures

All data collected on this protocol will be securely stored and managed in a REDCap database system at the University of California – San Francisco (UCSF) under the stewardship of the principal investigator.

This database will be developed and maintenance performed with support of the School of Medicine (SOM) at UCSF. REDCap was developed by Vanderbilt's CTSA and is currently used and supported by more than 1000 consortium partners. REDCap provides: 1) a stream-lined process for rapidly building a database; 2) an intuitive interface for collecting data (with data validation and audit trail); 3) automated export procedures for seamless data downloads to common statistical packages (SAS, SPSS, etc.); 4) branching logic, file uploading, and calculated fields; and 5) a quick and easy protocol set-up.

All connections to REDCap, both external and internal, occur over encrypted channels. Access to components of the system is role-based and can only be granted by administrators of the system. All collected information is stored on a standalone database server hosted by UCSF. The database server resides behind the UCSF internal firewall and access to the server is controlled via firewall rules.

All collected data is backed up daily, both on the local server and by the UCSF enterprise backup system.

12.4 Data Quality Control and Reporting

Weekly registration reports will be generated to monitor study participant accruals and completeness of registration data. Routine data quality reports will be generated to assess missing data and inconsistencies. Accrual rates and extent and accuracy of evaluations and follow-up will be monitored periodically throughout the study period and potential problems will be brought to the attention of the study team for discussion and action. The study team will conduct periodic random-sample data quality and protocol compliance audits.

12.5 Data and Safety Monitoring Plan

Level of Risk:

This study involves feasibility testing of a deprescription intervention with daily questionnaires that is believed to be overall low-risk.

Safety

Participants will be contacted via email or phone if they do not complete the daily symptom questionnaire for more than 3 consecutive days during their N-of-1 trial or if a dramatic worsening in their daily IPSS questionnaires are suggestive of increased risk of retention.

Other risk mitigation

The following procedures will be used to minimize risk and to ensure participant confidentiality both during and after the study:

- The eligibility criteria is a rigorous list of inclusionary/exclusionary criteria to minimize risk and was approved by physician PI Dr. Breyer.
- All study files, folders, and records will be kept in locked file cabinets that can be accessed only by study personnel.
- All data with PHI be securely exchanged in accordance with UCSF-approved policies.
- The study databases are housed in REDCap, a secure cloud data collection system behind IT-regulated and firewalls of the University of San Francisco, California. REDCap has the protections needed for storage of PHI and backup systems.
- Each participant will be assigned a unique numerical study identifier which will be used on study materials instead of names or other individually identifying information.
- Documents with participant identifiers (e.g., name) are stored on secure servers located behind a fire wall and accessed only by authorized study personnel.
- Information that could identify individual participants will not be released without written permission of the participant, except as necessary for monitoring by institutional review boards, the NIH, the Office for Human Research Protections, or other government agencies responsible for protecting participant safety.
- All investigators and support staff are HIPAA certified and have completed and are current with regard to IRB training.

13.0 PROTECTION OF HUMAN SUBJECTS

13.1 Informed Consent

Prior to the enrolment of each participant, the risks, benefits and objectives of the study will be reviewed with the participant. Alternative, non-protocol, treatment options will be discussed with the patient. Participants will be informed that participation in this clinical trial is voluntary and that the participant may withdraw consent at any time.

13.2 Potential Risks

Our eligibility criteria and screening procedures are established to exclude individuals for whom stopping tamsulosin may not be appropriate. Our eligibility criteria and screening procedures are established to exclude individuals for whom the study is not appropriate. Per the exclusion criteria, this includes patients with certain urologic conditions or psychiatric conditions. After obtaining

participant consent, the screening process will include verification of these factors by the clinician. This multi-gated comprehensive approach should systematically identify and screen out any individual for whom this study is contraindicated.

Risks of Research Participation – Participation in research involves some loss of privacy. We will do our best to make sure that all personal information gathered for this study is kept private. However, we cannot guarantee total privacy. There is also a minor risk to loss of confidentiality, either through the breach of data collected via the Internet, text messaging, or through the breach of secure study databases, physical files, etc. There is also some risk due to randomization. The intervention may be more burdensome and may not have a beneficial effect on their health outcomes compared to usual care.

13.3 Potential Benefits

There are no other direct benefits to the participants (financial compensation is purposely not presented as a benefit), except as to their feelings of being involved as participants in an important research study. The potential public health benefit to society in this study could be large, as we are targeting a common condition in older men (one in three develop LUTS/BPH in their lifetime). Scientific and clinical knowledge gained from this study could be extremely useful to practicing clinicians, individuals, policy makers, insurers, and public health planners. Additionally, study results may help individual patient decided if they should stay on tamsulosin.

This study will provide valuable insights into the feasibility of tamsulosin deprescription across a diverse patient population. The potential public health benefit to society in this study could be large, as we are targeting a common condition in older men (one in three older men develop LUTS/BPH in their lifetime). Our overarching goal is to build the evidence needed for a full-scale efficacy trial testing deprescription, thus this research has great potential to change the paradigms for LUTS/BPH management. Scientific and clinical knowledge gained from this study could be extremely useful to practicing clinician, individuals, policy makers, insurers, and public health planners developing interventions to prevent or treat LUTS/BPH. Thus, given the importance of knowledge to be gained and the anticipated benefit to research participants and others, the risks to subjects are reasonable.

13.4 Alternatives

We will have standard UCSF Institutional Review Board (IRB)-approved language on our consent form about other alternatives to participation. We will let participants know that they are free to choose not to participate in the study, and if they decide not to take part, there is no penalty to them. We will let them know they can participate in other research studies at UCSF (if available and if they are eligible) or they can choose not to participate in any research studies. We also encourage them to talk to their clinician about their choices before deciding to take part in the study.

13.5 Confidentiality

Every effort will be made to maintain patient confidentiality. Research and hospital records are confidential. Participants'' names and any other identifying information will not be used in reports or publications resulting from this study. Other authorized agencies and appropriate internal and external personnel may review patient records, as required by law. Only a participant ID number will identify all study participants on study documents. Additional participant confidentiality issues are covered in the participant consent.

13.6 Voluntariness of Research Participation

It is stated that taking part in this study is voluntary and participants have the right to withdraw at any time. Participation in the study will not impact on the clinical care participants receive.

13.7 Participant Privacy

Medical information is confidential. The participant's personal identity will not be used in reports that are written about the research. Every effort will be made to de-identify samples, reports, surveys whenever possible; and items will be physically labelled by an anonymous study-specific ID that is only linked to personal identifiers via a coded-document kept on secure computers, accessible only to study personnel. The results of any research using blood will not be placed in the medical record. The consent indicates that samples and genetic information collected may be shared with other qualified researchers. Such information will not include identifying information such as name.

14.0 STUDY MANAGEMENT

14.1 Pre-study Documentation

Before initiating this trial, the PI will have written and dated approval from the Institutional Review Board for the protocol, informed consent form, subject recruitment materials, and any other written information to be provided to participants before any protocol related procedures are performed on any participants.

14.2 Institutional Review Board Approval

The protocol, the proposed informed consent form, and all forms of participant-facing materials related to the study (e.g., advertisements used to recruit participants) will be reviewed and approved by the IRB. The initial protocol and all protocol amendments must be approved by the IRB prior to implementation.

14.3 Informed Consent

All participants must be provided a consent form describing the study with sufficient information for each participant to make an informed decision regarding their participation. The ICFs must be signed by the participant or the participant's legal representative before his participation in the study. The case history for each participant shall document the informed consent process and that written informed consent was obtained prior to participation in the study. A copy of each signed ICF must be provided to the participant or the patrician's legal representative. All signed and dated consent forms must remain in each participant's study file and must be available for verification by study monitors at any time.

The ICF should be revised whenever there are changes to procedures outlined in the informed consent or when new information becomes available that may affect the willingness of the participant to participate. Participants must be re-consented to the most current version of the consent forms during their participation in the study. For any updated or revised consent forms, the case history for each participant shall document the informed consent process and that written informed consent was obtained for the updated/revised consent form for continued participation in the study.

14.4 Changes in the Protocol

Once the protocol has been approved by the IRB, any changes to the protocol must be

documented in the form of an amendment. The amendment must be signed by the PI and approved by the IRB prior to implementation.

If it becomes necessary to alter the protocol to eliminate an immediate hazard to participants, an amendment may be implemented prior to IRB approval. In this circumstance, however, the PI must then notify the IRB according to institutional requirements.

14.5 Record Retention

The PI is required to prepare and maintain adequate and accurate case histories that record all observations and other data pertinent to the investigation on each study participant. Study documentation includes all CRFs, data correction forms or queries, source documents, Sponsor-Investigator correspondence, monitoring logs/letters, and regulatory documents (e.g., protocol and amendments, IRB correspondence and approval, signed participant consent forms). Source documents include all recordings of observations or notations of clinical activities and all reports and records necessary for the evaluation and reconstruction of the clinical research study. The PI shall retain records for a period of 2 years following the conclusion of the study.

14.6 Publications

The preparation and submittal for publication of manuscripts containing the study results shall be in accordance with a process determined by mutual written agreement among the Sponsor-Investigator and collaborators.

15.0 REFERENCES

1. Abrams P, Cardozo L, Fall M, et al. The standardisation of terminology in lower urinary tract function: report from the standardisation sub-committee of the International Continence Society. *Urology*. Jan 2003;61(1):37-49.
2. Wei JT, Calhoun E, Jacobsen SJ. Urologic diseases in america project: benign prostatic hyperplasia. *J Urol*. May 2008;179(5 Suppl):S75-80. doi:10.1016/j.juro.2008.03.141
3. Girman CJ, Jacobsen SJ, Tsukamoto T, et al. Health-related quality of life associated with lower urinary tract symptoms in four countries. *Urology*. Mar 1998;51(3):428-36. doi:10.1016/s0090-4295(97)00717-6
4. Blanker MH, Driessen LF, Bosch JL, et al. Health status and its correlates among Dutch community-dwelling older men with and without lower urogenital tract dysfunction. *European urology*. Jun 2002;41(6):602-7. doi:10.1016/s0302-2838(02)00172-0
5. Welch G, Weinger K, Barry MJ. Quality-of-life impact of lower urinary tract symptom severity: results from the Health Professionals Follow-up Study. *Urology*. Feb 2002;59(2):245-50.
6. Boyle P, Robertson C, Mazzetta C, et al. The relationship between lower urinary tract symptoms and health status: the UREPIK study. *BJU Int*. Oct 2003;92(6):575-80. doi:10.1046/j.1464-410x.2003.04448.x
7. Taylor BC, Wilt TJ, Fink HA, et al. Prevalence, severity, and health correlates of lower urinary tract symptoms among older men: the MrOS study. *Urology*. Oct 2006;68(4):804-9. doi:10.1016/j.urology.2006.04.019
8. Noguchi N, Chan L, Cumming RG, Blyth FM, Naganathan V. A systematic review of the association between lower urinary tract symptoms and falls, injuries, and fractures in community-dwelling older men. *The aging male : the official journal of the International Society for the Study of the Aging Male*. Sep 2016;19(3):168-174. doi:10.3109/13685538.2016.1169399
9. Bauer SR, Cawthon PM, Ensrud KE, et al. Lower urinary tract symptoms and incident functional limitations among older community-dwelling men. *Journal of the American Geriatrics Society*. Dec 24 2021;doi:10.1111/jgs.17633
10. Akerla J, Pesonen JS, Poyhonen A, et al. Impact of lower urinary tract symptoms on mortality: a 21-year follow-up among middle-aged and elderly Finnish men. *Prostate cancer and prostatic diseases*. Nov 8 2019;22:317–323. doi:10.1038/s41391-018-0108-z
11. Pesonen JS, Cartwright R, Vernooij RWM, et al. The Impact of Nocturia on Mortality: A Systematic Review and Meta-Analysis. *J Urol*. Jul 31 2019:101097ju0000000000000463. doi:10.1097/ju.0000000000000463
12. Saigal CS, Joyce G. Economic costs of benign prostatic hyperplasia in the private sector. *J Urol*. Apr 2005;173(4):1309-13. doi:10.1097/01.ju.0000152318.79184.6f
13. Malaeb BS, Yu X, McBean AM, Elliott SP. National trends in surgical therapy for benign prostatic hyperplasia in the United States (2000-2008). *Urology*. May 2012;79(5):1111-6. doi:10.1016/j.urology.2011.11.084
14. Bellinger AS, Elliott SP, Yang L, et al. Changes in initial expenditures for benign prostatic hyperplasia evaluation in the Medicare population: a comparison to overall Medicare inflation. *J Urol*. May 2012;187(5):1739-46. doi:10.1016/j.juro.2011.12.079
15. Oesterling JE. The origin and development of benign prostatic hyperplasia. An age-dependent process. *J Androl*. Nov-Dec 1991;12(6):348-55.
16. Lepor H. Pathophysiology of lower urinary tract symptoms in the aging male population. *Reviews in urology*. 2005;7 Suppl 7:S3-s11.
17. Miller M. Nocturnal polyuria in older people: pathophysiology and clinical implications. *Journal of the American Geriatrics Society*. Oct 2000;48(10):1321-9. doi:10.1111/j.1532-5415.2000.tb02608.x
18. D'Silva KA, Dahm P, Wong CL. Does this man with lower urinary tract symptoms have bladder outlet obstruction?: The Rational Clinical Examination: a systematic review. *JAMA : the journal of the American Medical Association*. Aug 06 2014;312(5):535-42. doi:10.1001/jama.2014.5555
19. Lerner LB, McVary KT, Barry MJ, et al. Management of Lower Urinary Tract Symptoms

Attributed to Benign Prostatic Hyperplasia: AUA GUIDELINE PART I-Initial Work-up and Medical Management. *J Urol*. Oct 2021;206(4):806-817. doi:10.1097/ju.0000000000002183
20. Welliver C, Feinstein L, Ward JB, et al. Trends in Lower Urinary Tract Symptoms Associated with Benign Prostatic Hyperplasia, 2004 to 2013: the Urologic Diseases in America Project. *J Urol*. Jan 2020;203(1):171-178. doi:10.1097/ju.0000000000000499
21. Yuan JQ, Mao C, Wong SY, et al. Comparative Effectiveness and Safety of Monodrug Therapies for Lower Urinary Tract Symptoms Associated With Benign Prostatic Hyperplasia: A Network Meta-analysis. *Medicine*. Jul 2015;94(27):e974. doi:10.1097/md.0000000000000974
22. Welk B, McArthur E, Ordon M, Anderson KK, Hayward J, Dixon S. Association of Suicidality and Depression With 5alpha-Reductase Inhibitors. *JAMA internal medicine*. May 01 2017;177(5):683-691. doi:10.1001/jamainternmed.2017.0089
23. Hagberg KW, Divan HA, Nickel JC, Jick SS. Risk of Incident Antidepressant-Treated Depression Associated with Use of 5alpha-Reductase Inhibitors Compared with Use of alpha-Blockers in Men with Benign Prostatic Hyperplasia: A Population-Based Study Using the Clinical Practice Research Datalink. *Pharmacotherapy*. May 2017;37(5):517-527. doi:10.1002/phar.1925
24. Unger JM, Till C, Thompson IM, Jr., et al. Long-term Consequences of Finasteride vs Placebo in the Prostate Cancer Prevention Trial. *J Natl Cancer Inst*. Dec 2016;108(12)doi:10.1093/jnci/djw168
25. Bird ST, Delaney JA, Brophy JM, Etminan M, Skeldon SC, Hartzema AG. Tamsulosin treatment for benign prostatic hyperplasia and risk of severe hypotension in men aged 40-85 years in the United States: risk window analyses using between and within patient methodology. *BMJ (Clinical research ed)*. Nov 5 2013;347:f6320. doi:10.1136/bmj.f6320
26. Oelke M, Becher K, Castro-Diaz D, et al. Appropriateness of oral drugs for long-term treatment of lower urinary tract symptoms in older persons: results of a systematic literature review and international consensus validation process (LUTS-FORTA 2014). *Age and ageing*. Sep 2015;44(5):745-55. doi:10.1093/ageing/afv077
27. Welk B, McArthur E, Fraser LA, et al. The risk of fall and fracture with the initiation of a prostate-selective alpha antagonist: a population based cohort study. *BMJ (Clinical research ed)*. Oct 26 2015;351:h5398. doi:10.1136/bmj.h5398
28. Press Y, Punchik B, Freud T. Orthostatic hypotension and drug therapy in patients at an outpatient comprehensive geriatric assessment unit. *Journal of hypertension*. Feb 2016;34(2):351-8. doi:10.1097/hjh.0000000000000781
29. Cindolo L, Pirozzi L, Fanizza C, et al. Drug adherence and clinical outcomes for patients under pharmacological therapy for lower urinary tract symptoms related to benign prostatic hyperplasia: population-based cohort study. *European urology*. Sep 2015;68(3):418-25. doi:10.1016/j.eururo.2014.11.006
30. Verhamme KM, Dieleman JP, Bleumink GS, Bosch JL, Stricker BH, Sturkenboom MC. Treatment strategies, patterns of drug use and treatment discontinuation in men with LUTS suggestive of benign prostatic hyperplasia: the Triumph project. *European urology*. Nov 2003;44(5):539-45.
31. Bauer SR, Scherzer R, Suskind AM, et al. Co-Occurrence of Lower Urinary Tract Symptoms and Frailty among Community-Dwelling Older Men. *Journal of the American Geriatrics Society*. Aug 21 2020;doi:10.1111/jgs.16766
32. Bauer SR, Jin C, Kamal P, Suskind AM. Association Between Lower Urinary Tract Symptoms and Frailty in Older Men Presenting for Urologic Care. *Urology*. Oct 10 2020;doi:10.1016/j.urology.2020.09.041
33. Parsons JK, Kashefi C. Physical activity, benign prostatic hyperplasia, and lower urinary tract symptoms. *European urology*. Jun 2008;53(6):1228-35. doi:10.1016/j.eururo.2008.02.019
34. Yokoyama T, Watanabe T, Saika T, et al. Natural course of lower urinary tract symptoms following discontinuation of alpha-1-adrenergic blockers in patients with benign prostatic hyperplasia. *International journal of urology : official journal of the Japanese Urological Association*. 2007/7// 2007;14(7):598-601. doi:10.1111/J.1442-2042.2007.01784.X
35. Chung JH, Lee JY, Kang DH, et al. Evaluation of patient outcome after discontinuation of alfuzosin treatment for benign prostatic hyperplasia: a multicentre, prospective study. *International*

journal of clinical practice. 2013/9// 2013;67(9):870-875. doi:10.1111/IJCP.12108
36. Barkin J, Guimarães M, Jacobi G, Pushkar D, Taylor S, Van Vierssen Trip OB. Alpha-blocker therapy can be withdrawn in the majority of men following initial combination therapy with the dual 5α-reductase inhibitor dutasteride. *European Urology*. 2003/10// 2003;44(4):461-466. doi:10.1016/S0302-2838(03)00367-1
37. O'Mahony D, O'Sullivan D, Byrne S, O'Connor MN, Ryan C, Gallagher P. STOPP/START criteria for potentially inappropriate prescribing in older people: version 2. *Age and ageing*. 2015/3// 2015;44(2):213-218. doi:10.1093/AGEING/AFU145
38. Fick DM, Semla TP, Steinman M, et al. American Geriatrics Society 2019 Updated AGS Beers Criteria® for Potentially Inappropriate Medication Use in Older Adults. *Journal of the American Geriatrics Society*. 2019/4// 2019;67(4):674-694. doi:10.1111/JGS.15767
39. Renoncourt T, Saint F, Bennis Y, Mondet L, Bloch F. Potentially Inappropriate Prescribing for Prostatic Hyperplasia in Older Persons. *Journal of the American Medical Directors Association*. 2022/6// 2022;23(6):992-997. doi:10.1016/J.JAMDA.2021.09.023
40. Edelman M, Jellema P, Hak E, Denig P, Blanker MH. Patients' Attitudes Towards Deprescribing Alpha-Blockers and Their Willingness to Participate in a Discontinuation Trial. *Drugs & aging*. 2019/12// 2019;36(12):1133-1139. doi:10.1007/S40266-019-00712-6
41. Barry MJ, Fowler FJ, Jr., O'Leary MP, et al. The American Urological Association symptom index for benign prostatic hyperplasia. The Measurement Committee of the American Urological Association. *J Urol*. Nov 1992;148(5):1549-57; discussion 1564.
42. Barry MJ, Williford WO, Fowler FJ, Jr., Jones KM, Lepor H. Filling and voiding symptoms in the American Urological Association symptom index: the value of their distinction in a Veterans Affairs randomized trial of medical therapy in men with a clinical diagnosis of benign prostatic hyperplasia. *J Urol*. Nov 2000;164(5):1559-64.
43. Barry MJ, Williford WO, Chang Y, et al. Benign prostatic hyperplasia specific health status measures in clinical research: how much change in the American Urological Association symptom index and the benign prostatic hyperplasia impact index is perceptible to patients? *J Urol*. Nov 1995;154(5):1770-4.
44. Reeve E, Low L-F, Shakib S, Hilmer SN. Development and validation of the revised patients' attitudes towards deprescribing (rPATD) questionnaire: versions for older adults and caregivers. *Drugs & aging*. 2016;33:913-928.
45. Voils CI, Gavin KL, Thorpe CT, et al. Validating a Self-Reported Medication Nonadherence Measure in the Context of Multiple Chronic Diseases and Routes of Medication Administration Among Patients with Type 2 Diabetes. *Patient preference and adherence*. 2022:3119-3130.
46. Kravitz RL, Schmid CH, Marois M, et al. Effect of Mobile device–supported single-patient multi-crossover trials on treatment of chronic musculoskeletal pain: A randomized clinical trial. *JAMA internal medicine*. 2018;178(10):1368-1377.
47. Bhuyan SS, Lu N, Chandak A, et al. Use of mobile health applications for health-seeking behavior among US adults. *Journal of medical systems*. 2016;40:1-8.
48. Moore CG, Carter RE, Nietert PJ, Stewart PW. Recommendations for planning pilot studies in clinical and translational research. *Clinical and translational science*. 2011;4(5):332-337.
49. Viechtbauer W, Smits L, Kotz D, et al. A simple formula for the calculation of sample size in pilot studies. *Journal of clinical epidemiology*. 2015;68(11):1375-1379.
