# Supplementary material for: Tamsulosin Deprescribing for Lower Urinary Tract Symptoms in Older Men: A Randomized Clinical Trial
Source: JAMA Netw Open. 2026 Jul 6;9(7):e2621639. doi: 10.1001/jamanetworkopen.2026.21639 (PMC13338806; doi:10.1001/jamanetworkopen.2026.21639)
Supplement: Supplement 3. — Data Sharing Statement [file jamanetwopen-e2621639-s003.pdf]

Data Sharing Statement

Bauer. Tamsulosin Deprescribing for Lower Urinary Tract Symptoms in Older Men. *JAMA Netw Open*. Published July 06, 2026. doi:10.1001/jamanetworkopen.2026.21639

Data
Additional Information: NCT05415748
Data available: No
